# Cellular mechanisms of taste disturbance induced by the non-steroidal anti-inflammatory drug, diclofenac, in mice

**DOI:** 10.3389/fncel.2023.1279059

**Published:** 2023-12-18

**Authors:** Ayaka Hirayama, Shusuke Iwata, Asami Oike, Yuko Kawabata, Yuki Nagasato, Shingo Takai, Keisuke Sanematsu, Ichiro Takahashi, Noriatsu Shigemura

**Affiliations:** ^1^Section of Oral Neuroscience, Graduate School of Dental Science, Kyushu University, Fukuoka, Japan; ^2^Section of Orthodontics and Dentofacial Orthopedics, Division of Oral Health, Growth and Development, Kyushu University, Fukuoka, Japan; ^3^Research and Development Center for Five-Sense Devices, Kyushu University, Fukuoka, Japan; ^4^Oral Health/Brain Health/Total Health Research Center, Faculty of Dental Science, Kyushu University, Fukuoka, Japan

**Keywords:** taste disorder, NSAIDs, diclofenac, COX-1, cytosolic prostaglandin E synthase

## Abstract

Drug-induced taste disorders are a serious problem in an aging society. This study investigated the mechanisms underlying taste disturbances induced by diclofenac, a non-steroidal anti-inflammatory drug that reduces pain and inflammation by inhibiting the synthesis of prostaglandins by cyclooxygenase enzymes (COX-1 and COX-2). RT-PCR analyses demonstrated the expression of genes encoding arachidonic acid pathway components such as COX-1, COX-2 and prostaglandin synthases in a subset of mouse taste bud cells. Double-staining immunohistochemistry revealed that COX-1 and cytosolic prostaglandin E synthase (cPGES) were co-expressed with taste receptor type-1 member-3 (T1R3), a sweet/umami receptor component, or gustducin, a bitter/sweet/umami-related G protein, in a subset of taste bud cells. Long-term administration of diclofenac reduced the expression of genes encoding COX-1, gustducin and cPGES in mouse taste buds and suppressed both the behavioral and taste nerve responses to sweet and umami taste stimuli but not to other tastants. Furthermore, diclofenac also suppressed the responses of both mouse and human sweet taste receptors (T1R2/T1R3, expressed in HEK293 cells) to sweet taste stimuli. These results suggest that diclofenac may suppress the activation of sweet and umami taste cells acutely via a direct action on T1R2/T1R3 and chronically via inhibition of the COX/prostaglandin synthase pathway inducing down-regulated expression of sweet/umami responsive components. This dual inhibition mechanism may underlie diclofenac-induced taste alterations in humans.

## 1 Introduction

The sense of taste has an important influence on food preferences and appetite. Drug-induced taste disorder is an adverse effect of some medications that can lead to a reduction in quality of life, loss of appetite, decreased food intake and malnutrition. Non-steroidal anti-inflammatory drugs (NSAIDs) are widely used to reduce pain and inflammation and are included in the World Health Organization’s Model List of Essential Medicines. Although NSAIDs are associated with various adverse effects including taste disturbances (Schiffman et al., 2000; Lipton et al., 2020), the molecular mechanisms of NSAID-induced taste disorder remain largely unknown.

Diclofenac is a commonly prescribed NSAID with anti-inflammatory, analgesic and anti-pyretic properties, and this drug is used to treat a variety of conditions associated with acute and chronic pain and/or inflammation (Gan, 2010). Diclofenac inhibits both the COX-1 and COX-2 isoforms of cyclooxygenase (also known as prostaglandin H synthase), thereby reducing the synthesis of prostanoids such as prostaglandins E2 (PGE2), D2 (PGD2), and F2 (PGF2), prostacyclin (PGI2) and thromboxane A2 (TXA2) (Ku et al., 1986; Vane et al., 1998). PGE2 is the major prostanoid produced in inflammation, and inhibition of its synthesis is believed to be the primary mechanism underlying the potent analgesic and anti-inflammatory properties of NSAIDs (Patrono et al., 2001; Grosser et al., 2006).

Taste buds in the epithelium of the tongue and palate consist of 50–100 taste cells innervated by gustatory nerves. Recent molecular biology studies have identified the taste receptors for the five basic tastes as well as the downstream taste-associated molecules expressed in taste cells (Lindemann, 2001; Chandrashekar et al., 2006; Shigemura and Ninomiya, 2016). There are two main types of taste receptor: G protein-coupled receptors, which include the sweet taste receptor (a heterodimer of taste receptor type 1 member 2 [T1R2] and T1R3), bitter taste receptor (type 2 taste receptor, T2R) and umami taste receptor (a heterodimer of T1R1 and T1R3); and channel receptors, which include the salty taste receptor (epithelial sodium channel [ENaCα]) and sour taste receptor (otopetrin-1 [Otop1]). Each taste receptor is expressed in an independent group of taste cells, and most taste cells are tuned to respond to a single taste quality. T1R- or TAS2R-positive cells express common signaling pathway molecules such as gustducin (a G-protein), phospholipase C-β2, inositol 1,4,5-triphosphate receptor type 3, and transient receptor potential melastatin-5 (TRPM5). By contrast, Otop1-expressing cells specifically express carbonic anhydrase-4 (CA4) but do not express phospholipase C-β2 or TRPM5. Additionally, taste bud cells have been classified into four main types based on electron microscopy observations: type I, type II, type III and basal (undifferentiated) cells. Type I cells express nucleoside triphosphate diphosphohydrolase-2 (NTPDase-2), which hydrolyzes extracellular ATP; type II cells express T1Rs, T2Rs and associated signaling pathway molecules; and type III cells express Otop1 and CA4 (He et al., 2004; Chandrashekar et al., 2009; Chaudhari and Roper, 2010; Tu et al., 2018).

A previous study reported that COX-2, monoglyceride lipase and group IIA phospholipase A_2_ (PLA2-IIa) were co-expressed with TRPM5 in a subset of taste bud cells of the rat. The authors also demonstrated that TRPM5 was activated by the application of arachidonic acid in a HEK293 cell heterologous expression system (Oike et al., 2006). Comprehensive expression analysis using RNA sequencing has also revealed the expression of these arachidonic acid pathway genes in each type of mouse taste cell (Sukumaran et al., 2017). However, the mechanism by which diclofenac affects taste sensitivity *in vivo* and *in vitro* and whether this involves COX enzymes remain unclear.

We hypothesized that COX-1, COX-2 and prostaglandin synthases are expressed in taste buds and involved in the mechanisms underlying the adverse effects of diclofenac on the taste system. NSAIDs are used both short- and long-term, according to clinical need. Therefore, we first investigated the expressions of COX-1, COX-2 and arachidonic acid pathway-related molecules in mouse taste buds. Then, we examined the effects of administering diclofenac sodium for 30 days (“long-term administration”) on the expression of target molecule genes as well as on the behavioral and taste nerve responses of mice to taste stimuli. Finally, we performed Ca^2+^ imaging experiments to determine the effects of a single administration of diclofenac (“short-term administration”) on the activation of mouse and human sweet taste receptors (T1R2/T1R3) expressed in HEK293 cells.

## 2 Materials and methods

All experiments and procedures were performed according to the National Institutes of Health *Guide for the Care and Use of Laboratory Animals* and approved by the committee for Laboratory Animal Care and Use at Kyushu University, Japan (approval no. A21-002-0, 14 September 2020).

### 2.1 Animals

The experimental subjects were adult male and female C57BL/6NCrj (B6) mice (age, 8–12 weeks; weight, 20–32 g; Charles River, Tokyo, Japan). Animals were maintained in a constant room temperature of 24 ± 1°C under a light/dark cycle (12:12 h, lights on at 08:00 am) with *ad libitum* access to water and food. Both male and female mice were used for the experiments. Diclofenac (1.5 mg/kg body weight) or saline (control) were given by intraperitoneal injection every day for 30 days.

Diclofenac, an inhibitor of COX, is one of the most widely used NSAIDs. Diclofenac is often used short-term or long-term as an anti-pyretic and analgesic drug. It has been reported that taste bud cells are continually renewed with an average lifespan of 10–14 days (Beidler and Smallman, 1965; Farbman, 1980). Thus, a taste bud contains a mixture of cells at different stages of differentiation. Given the turnover cycle of 10–14 days, taste bud cells would be expected to be fully affected by diclofenac after 30 days of treatment. Therefore, daily treatment for 30 days was used for the long-term administration experiments.

### 2.2 Drugs and taste compounds

Diclofenac was dissolved in saline (0.9% NaCl). The drug concentrations were adjusted to provide the appropriate dose in a total volume of 1.0 mL. In this study, the dose of diclofenac administered to mice (1.5 mg/kg body weight) was determined by reference to the human dose indicated in the prescribing information [75–100 mg per day (1.25–1.7 mg/kg body weight)].

The taste solutions used in this study were: NaCl, sucrose (Suc) [with or without quinine hydrochloride (QHCl)], glucose (Glc), saccharin (Sac), QHCl, NH_4_Cl (all purchased from Fujifilm Wako Pure Chemical Corporation, Osaka, Japan) and monopotassium glutamate (MPG, Sigma-Aldrich, St Louis, MO, USA). All taste solutions were dissolved in distilled water (DW).

### 2.3 Reverse transcription-polymerase chain reaction (RT-PCR)

Reverse transcription-polymerase chain reaction was conducted as described previously (Shigemura et al., 2004, 2013; Yoshida et al., 2010; Oike et al., 2022). The tongues were dissected from B6 mice (*n* = 3) and divided into anterior and posterior parts, which were injected with 0.3 and 0.1 mL dispase α (0.1 U/mL; Sigma-Aldrich, St Louis, MO, USA), respectively, and incubated for 10 min at 37°C in phosphate-buffered saline (PBS). Taste buds were isolated from the fungiform papillae (FP) and circumvallate papillae (CV) of each mouse using elastase (0.28 U/mL; Elastin Products Company, Owensville, MO, USA), aspirated with a transfer pipette, and pooled. The RNeasy Plus Micro kit (Qiagen) was used to purify RNAs pooled from 40 taste buds from the FP and CV, respectively, or a 1 mm × 1 mm block of the adjacent epithelial tissue without taste buds (Non-taste tissue: collected from the underside of the tongue). cDNAs were generated by RT [oligo(dT)12–18 primer] using the superscript pre-amplification system (Invitrogen, Carlsbad, CA, USA). The PCR was carried out under the following conditions: 95°C for 15 min (1 cycle); 94°C for 30 s, 55°C for 30 s and 72°C for 36 s (35 cycles). Each 20 μL of PCR solution contained 0.5 U of TaqDNA polymerase (TaKaRa Ex TaqHS; Takara Bio, Kusatsu, Japan), 2 μL of 10 × PCR buffer containing 20 mM Mg^2+^, 0.2 mM of each dNTP and 0.6 μM of each primer pair. The resulting amplification products were visualized in a 2% agarose gel with 0.5 μg/mL ethidium bromide. The primer sequences are shown in Table 1. To determine the signals from genomic DNA, purified RNA samples were treated in parallel with or without reverse transcriptase. RT-PCR amplicons were sequenced.

**TABLE 1:** Primer used for RT-PCR.

Gene	Forward primer	Reverse primer	Product size	Accession No.
*Gapdh*	TGTGTCCGTCGTGGATCTGA	TTGCTGTTGAAGTCGCAGGAG	150	NM_001289726
*Gustducin*	TGCTTTGAAGGAGTGACGTG	GTAGCGCAGGTCATGTGAGA	341	NM_001081143
*Ptgs1 (COX-1)*	CCTCACCAGTCAATCCCTGT	CTGGTGGGTGAAGTGTTGTG	536	NM_008969
*Ptgs2 (COX-2)*	GCGAGCTAAGAGCTTCAGGA	GAGAAGGCTTCCCAGCTTTT	498	NM_011198
*mPges1*	GCTGCGGAAGAAGGCTTTTG	GGGACTTCATCTAGCATCCCA	538	NM_022415
*cPges*	CGCACGCACGCTCCTT	TGAGCCAATTAAGCTTTGCCC	553	NM_019766
*cPLA2*	GCCTCTCTTCACGTGTCTCC	GACCCAACTTGCTTGGTTGT	418	NM_001305632
*sPLA2*	ACCACCATCCAAGAGAGCTG	CTTTTCAGCATTTGGGCTTC	470	NM_001082531
*Pgds*	TCCGGGAGAAGAAAGCTGTA	CCAGCCCTCTGACTGACTTC	439	NM_001420744
*Pgis*	GCAGCCTGTTTCACAGATGA	CAGGTCGAAATGAGTCAGCA	430	NM_001420752
*Tbxas1*	CCCTGTCCTCTTCTGAGTGC	GCCTCTGCTGTGAACCTTTC	472	NM_001410475
*Prxl2b*	CGTAGGAGCGTGTGTCTTGA	CCTCCCACACACCTCTTCAT	580	NM_025582

### 2.4 Immunohistochemistry

Details of the procedures used for immunohistochemistry are described in our previous publications (Takai et al., 2015; Oike et al., 2022). The dissected tongues of B6 mice [*n* = 10 (5 males and 5 females)] were fixed in 4% paraformaldehyde in PBS for 45 min at 4°C and then dehydrated with sucrose solutions (10% for 1 h, 20% for 1 h, and 30% for 3 h at 4°C). A frozen block of tongue tissue was embedded in optimal-cutting-temperature compound (Sakura Finetechnical, Tokyo, Japan) and sectioned into 10-μm-thick slices, which were mounted on silane-coated glass slides and air-dried. The sections were washed with Tris/NaCl/Tween (TNT) buffer, treated with Blocking One solution for 1 h (Nacalai Tesque, Kyoto, Japan), and incubated with the primary antibodies overnight at 4°C. After washing with TNT buffer, the sections were incubated with secondary antibodies for 2 h and then washed with TNT. The immunofluorescence of labeled cells was detected using a laser-scanning microscope (FV-1000, Olympus, Tokyo, Japan), and images were captured using Fluoview software (Olympus, Tokyo, Japan). Cells expressing COX-1, cPGES, T1R3, gustducin and CA4 were considered positive if their signal density was greater than the mean + 2 standard deviations of the signal density of taste cells without primary antibody (negative control). The same cells found in consecutive sections were counted only once. The primary antibodies were rabbit anti-COX-1 (1:100; Abcam, Cambridge, UK), mouse anti-cytosolic PGE synthase (cPGES; 1:100; Abcam, Cambridge, UK), goat anti-T1R3 (1:100; Santa Cruz Biotechnology, Dallas, TX, USA), custom-made guinea pig anti-Gα-gustducin (residues 95–106, 13 amino acids, Cys-YVNPRSREDQEQ-OH; 1:1000), and goat anti-CA4 (1:100; R&D Systems, Minneapolis, MN, USA). The secondary antibodies were Alexa Fluor 488 donkey anti-rabbit IgG (1:300; Invitrogen, Waltham, MA, USA) for immunostaining of COX-1, Alexa Fluor 488 donkey anti-mouse IgG (1:300; Invitrogen, USA) for immunostaining of cPGES, Alexa Fluor 568 donkey anti-goat IgG (1:300; Invitrogen, USA) for immunostaining of T1R3 and CA4, and Alexa Fluor 647 donkey anti-guinea pig IgG (1:200; Invitrogen, USA) for immunostaining of gustducin.

### 2.5 Behavioral test

We used short-term (10 s) tests to study the effects of diclofenac on behavioral responses. Details of the procedures used for this test are described in our previous publications (Murata et al., 2003; Yoshida et al., 2010; Shigemura et al., 2013; Takai et al., 2015; Oike et al., 2022). Each B6 mouse with 23 h water deprivation was placed in a test box on day 1 of training and given free access to DW during a 1 h session. The licks were detected by a lick meter equipped with a laser beam lick sensor (Yutaka Electronics, Nagoya, Japan) and recorded on a strip chart recorder. Days 2–5 were the training session. During this period, the mice were trained to drink DW on an interval schedule, consisting of 10 s periods of presentation of the DW alternated with 20 s intervals in the 1 h session. From day 6, the number of licks for each test stimulus and for DW were counted during the first 10 s after the mouse’s first lick.

Measurements of the number of licks were made for mice who had received intraperitoneal injections of either vehicle (physiological saline) once every morning for 30 days or diclofenac (1.5 mg/kg body weight) dissolved in vehicle once every morning for 30 days. The DW-drinking training session took place at the end of the 30-days period, and daily diclofenac administration continued during the subsequent test session. On each test day, the first test stimulus given to the mouse was DW, and then the following solutions were tested in a randomized order: 10–1000 mM Suc with 0.5 mM QHCl, 0.1–20 mM Sac with 0.5 mM QHCl, 1–1000 mM MPG with 0.5 mM QHCl, 0.01–1 mM QHCl, 10–1000 mM NaCl, 1–30 mM HCl, and 10–1000 KCl. A total of 0.5 mM QHCl was added to each Suc, Sac and MPG test solution to enable concentration-dependent changes in the lick rates for sweeteners and umami to be determined more clearly. The mouse could initiate up to 40 test trials (randomized multiple solutions including DW rinse) per day, which were separated by a 20 s interval. The test session lasted for four days. All solutions and concentrations were tested once a day per mouse. The mean value of the lick counts for each test stimulus was calculated for each mouse.

### 2.6 Chorda tympani (CT) nerve recording

Gustatory nerve responses to the lingual application of taste solutions were recorded from the CT nerve as described previously (Yoshida et al., 2010; Shigemura et al., 2013; Takai et al., 2015). All procedures were performed under pentobarbital anesthesia (50–60 mg/kg body weight). Each B6 mouse (long-term administration; *n* = 6–15 control mice, *n* = 6–13 treated mice; single administration; *n* = 5–7 control mice, *n* = 5–7 treated mice) was fixed in the supine position with its head in a holder to allow dissection of the CT nerve. The right CT nerve was freed from the surrounding tissues after removal of the pterygoid muscle and cut at the point of its entry to the bulla. The entire nerve was placed on an Ag/AgCl electrode, and indifferent electrodes were placed in nearby tissue. Neural activity was fed into an amplifier (K-1; Iyodenshikagaku, Nagoya, Japan) and monitored on an oscilloscope and audio monitor. Whole nerve responses were integrated with a time constant of 1.0 s and recorded on a computer using a PowerLab/sp4 system (AD Instruments, Bella Vista, NSW, Australia). The anterior half of the tongue was enclosed in a silicone rubber flow chamber to allow stimulation of the FP. Taste solutions (100 mM NH_4_Cl, 10–1000 mM Glc, 10–1000 mM Suc, 0.1–20 mM Sac, 10–1000 mM MPG, 0.1–10 mM QHCl, 10–1000 mM NaCl, 0.1–10 mM HCl) were delivered to each part of the tongue by gravity flow for 30 s. The tongue was washed with DW for ∼60 s between successive stimulations. Only stably recorded data were used in the analysis. The magnitude of the integrated whole nerve response was measured during a 30 s period of stimulation. The response was averaged over a 20 s period after excluding the data for the initial and final 5 s periods, and this value was normalized to the response to 100 mM NH_4_Cl to account for inter-animal variations in the absolute responses. In the long-term administration experiments, mice were treated with diclofenac daily for a period of 30 days. For the single dose experiments, recording of the CT nerve response commenced immediately after the administration of diclofenac sodium.

### 2.7 Quantitative PCR (qPCR)

Total RNA extraction from pooled taste buds was conducted as described above for RT-PCR (*n* = 3–6 control mice, *n* = 3–6 treated mice). The RNA concentration was measured using a NanoDrop ND-1000 spectrophotometer (Thermo Fisher Scientific, Waltham, MA, USA). Fast SYBR Green Master Mix (Applied Biosystems, CA, USA) was used for qPCR. PCR was performed as follows: 50°C for 2 min and 95°C for 2 min (1 cycle); 95°C for 3 s and 60°C for 30 s (40 cycles); and 95°C for 15 s, 60°C for 1 min, and 95°C for 15 s (1 cycle for melting curve analysis). StepOnePlus software v2.3 (Applied Biosystems, Waltham, MA, USA) was used for data analysis. Data were obtained from at least three independent experiments, and all reactions were performed in duplicate. The presence of a single amplicon was confirmed by melting curve analysis and agarose gel electrophoresis. The qPCR data were normalized using the ΔΔCt method with glyceraldehyde-3-phosphate dehydrogenase (*Gapdh*) in each sample as the reference (Takai et al., 2015). All primer pairs were chosen so that each primer originated from a different exon. The qPCR primer sequences are shown in Table 2. Details of the procedures used for qPCR are described in our previous publication (Oike et al., 2022).

**TABLE 2:** Primer used for q-PCR.

Gene	Forward primer	Reverse primer	Product size	Accession No.
*Gapdh*	TGTGTCCGTCGTGGATCTGA	TTGCTGTTGAAGTCGCAGGAG	150	NM_001289726
*NTPDase*	ATGGCTGGAAAGTTGGTGTCA	TCTTGGGTAGGGACGCACA	92	NM_009849
*Gustducin*	AGGGCATCTGAATACCAGCTCAA	CTGATCTCTGGCCACCTACATCAA	196	NM_001081143
*T1R3*	CAAGGCCTGCAGTGCACAA	AGGCCTTAGGTGGGCATAATAGGA	92	NM_031872
*CA4*	TACTGAAGACTCAGGCTGGTG	GGTCATAGCCGACGAGGATG	179	NM_007607
*Krt8*	TGAACAACAAGTTCGCCTCCTT	CCAGAGATTGGAGTTGTTCTTGT	122	NM_001406028
*Ptgs1 (COX-1)*	CCAGAGTCATGAGTCGAAGGA	CCTGGTTCTGGCACGGATAG	147	NM_008969
*Ptgs2 (COX-2)*	AAGCGAGGACCTGGGTTCA	GGGGATACACCTCTCCACCA	148	NM_011198
*mPges1*	AGATGAGGCTGCGGAAGAAG	TTGCGGTGGGCTCTGAGG	115	NM_022415
*mPges2*	CCTCCTACAGGAAAGTGCCC	CAGGGGCTGCCCTGAAAC	119	NM_133783
*cPges*	GCCAGTCATGGCCTAGGTT	CACCCATGTGATCCATCATCTCAG	147	NM_019766

### 2.8 Single-cell Ca^2+^ imaging

We used single-cell calcium imaging to investigate the effects of diclofenac sodium on sweet taste receptors. The details of the procedure are described in our previous publications (Sanematsu et al., 2014, 2016, 2023) First, the cells were transfected in 35 mm recording chambers for 24 h. Subsequently, they were loaded with 3.0 mM Fluo-4 acetoxymethyl ester (Invitrogen) for 3 min at 37°C. To wash the cells, Hank’s balanced salt solution (HBSS; Thermo Fisher Scientific, USA) was used. The activation kinetics of the dye-loaded cells were measured using a bath perfusion system. The taste solution was diluted in HBSS solution containing 10 mM HEPES and then sequentially flown into the cells for 25 s at a flow rate of 1.0 mL/min. To prevent desensitization of the cells caused by the prior taste solution, each taste solution was applied at 5-min intervals. We used a S Fluor 620/0.75 objective lens attached to a TE300 microscope made by Nikon in Tokyo, Japan. A cooled CCD camera (C6790) from Hamamatsu Photonics K.K. in Shizuoka, Japan was used to capture fluorescence images with an imaging speed of 2 s per capture. The AquaCosmos software (version 1.3, Hamamatsu Photonics K.K.) was utilized to obtain and analyze fluorescence images. Background subtraction was performed. The changes in intracellular Ca^2+^ in individual cells were monitored as changes in the fluorescence of fluo-4. Fluorometric signals were expressed as relative changes in fluorescence: ΔF/F_0_ = (F-F_0_)/F_0_, where F_0_ denotes the baseline fluorescence level. Baseline was determined at 30 frames before any stimulus was given. The final criteria for the occurrence of a response were the following: F value was larger than the mean plus two standard deviations of the baseline. Ca^2+^ changes were measured and averaged from 5 to 25 s after stimulus onset. The data are expressed as the mean ± S.E. of the ΔF/F_0_ value. We categorized cells that responded at least three times to sweet-taste stimuli as responding cells and measured their intracellular Ca^2+^ changes, while excluding the other cells. In a single experiment, we measured cell responses to four different reagents, namely 0.3 mM SC, 30 mM Cyc, 10 mM Sac, and 10 mM Sac + Dic (at 3–4 different concentrations).

### 2.9 Statistical analysis

All data are shown as the mean ± standard error of the mean (SEM). Data for the behavioral and taste nerve recording tests were compared using factorial two-way ANOVA and *post-hoc* Student’s *t*-tests. Data for the Ca^2+^ imaging experiments were compared using one-way ANOVA and *post-hoc* Tukey’s tests. Data for the other experiments were compared using Student’s *t*-tests. Statistical comparisons were made using SPSS Statistics 19 (IBM, Armonk, NY, USA). *P* < 0.05 was taken to indicate statistical significance.

## 3 Results

### 3.1 mRNAs for arachidonic acid pathway-related molecules including *Ptgs1* (COX-1) and *Ptgs2* (COX-2) were expressed in mouse taste buds

**FIGURE 1:**
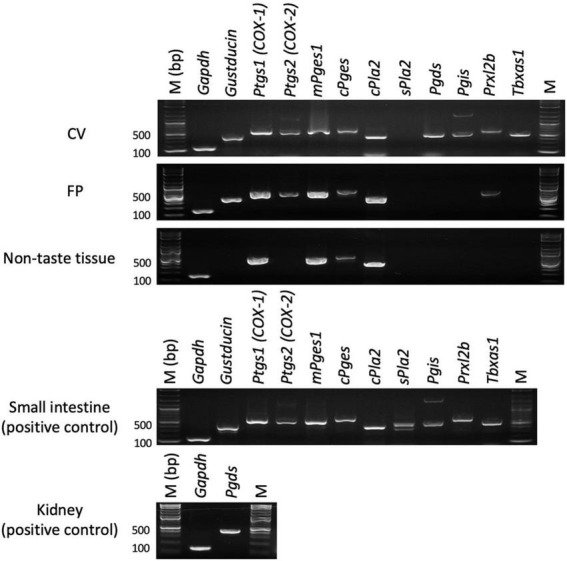
Reverse transcription-polymerase chain reaction was used to amplify the mRNAs for arachidonic acid pathway component molecules in the circumvallate papillae (CV), fungiform papillae (FP), tongue non-taste epithelial tissue, small intestine and lung (positive control for arachidonic acid pathway components) of B6 mice. Arachidonic acid pathway components: prostaglandin-endoperoxide synthase-1/2 (*Ptgs1/Ptgs2*), microsomal/cytosolic prostaglandin E synthase (*mPges1/cPges*), cytosolic/secretory phospholipase A2 (*c/s Pla2*), prostaglandin D2 synthase (*Pgds*), prostaglandin I2 synthase (*Pgis*), peroxiredoxin-like 2B (*Prxl2b*), thromboxane A synthase 1 (*Tbxas1*). *Gustducin* (a taste cell marker), glyceraldehyde-3-phosphate dehydrogenase (*Gapdh*, a housekeeping gene), M (bp) 100 bp marker ladder. Uncropped blots can be found in Supplementary Figure 1.

First, we performed RT-PCR experiments to investigate the expressions of genes encoding COX-1, COX-2 and arachidonic acid pathway-related molecules in the taste buds (CV and FP), non-taste epithelial tissue (ET, which does not contain taste buds), small intestine and lung (positive control tissues for arachidonic acid pathway-related molecules) of B6 mice (Figure 1). PCR bands of the correct sizes for *Ptgs1* (536 bp) and *Ptgs2* (498 bp), which correspond to COX-1 and COX-2, respectively, were detected in the taste tissues (CV and FP). In addition, expression of the genes encoding PGD2 synthase (*Pgds*), PGI2 synthase (*Pgis*), peroxiredoxin-like 2B (*Prxl2b*) and TXA synthase-1 (*Tbxas1*), which are components of the arachidonic acid pathway, was detected in the taste tissues (CV and FP) but not ET. Expression of the genes for COX-1 (*Ptgs1*), cytosolic PLA2 (*Pla2g4a*) and PGE synthases (*mPges1* and *cPges*) were observed not only in the taste tissues but also in ET, whereas secretory PLA2 (*Pla2g2a*) was not expressed in the FP, CV, or ET. These results suggest that mouse taste bud cells may contain a functional arachidonic acid pathway that synthesizes prostaglandins. In positive control experiments, an RT-PCR product of the correct size for gustducin (341 bp), a marker of type II taste cells, was found in both CV and FP taste buds but not in ET. *Gapdh* mRNA (150 bp) was detected in all tissues. All control experiments in which the reverse transcriptase enzyme was omitted (RT-) yielded negative results.

### 3.2 COX-1 was expressed mainly in type II taste cells

**FIGURE 2:**
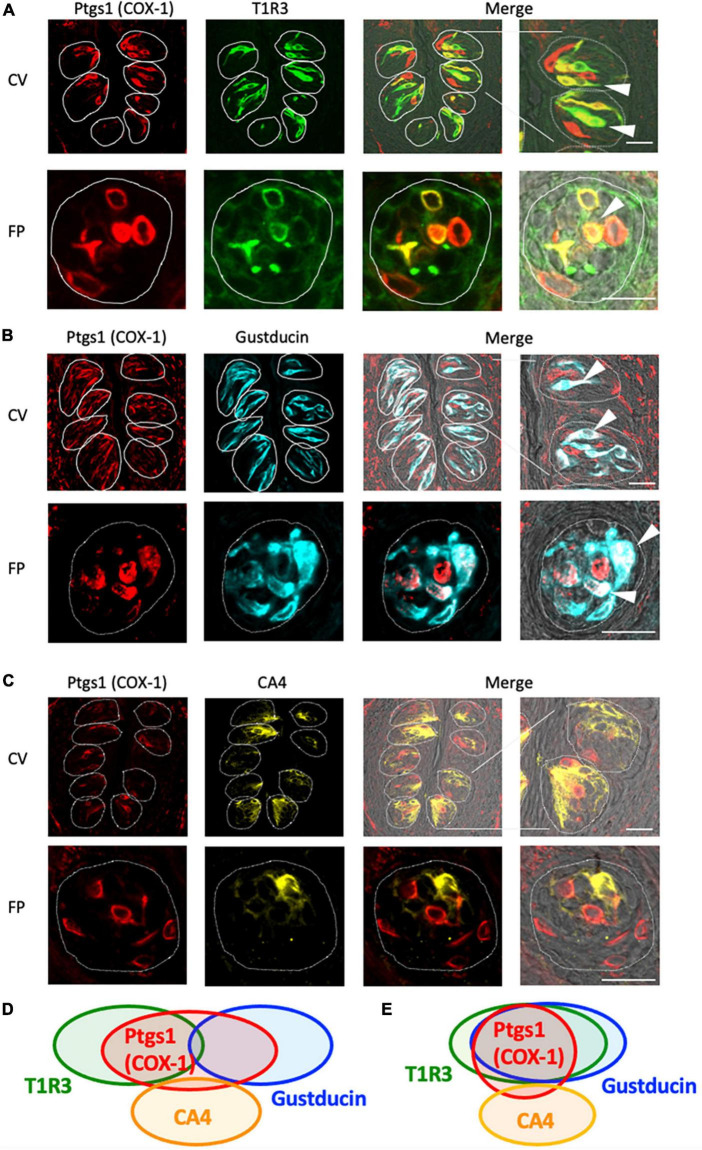
Prostaglandin-endoperoxide synthase-1 (COX-1) expression in the circumvallate papillae (CV) and fungiform papillae (FP) of mice. **(A,B)** COX-1 immunoreactivity (red) in the CV and FP was observed mainly in taste bud type II cells. Arrowheads denote COX-1 + T1R3 (green) double-positive cells (yellow) **(A)**, and COX-1 + gustducin (magenta) double-positive cells (white) **(B)**. **(C)** A few COX-1-positive cells expressed carbonic anhydrase-4 (CA4, yellow), a marker of taste bud type III cells. Dotted lines outline individual taste buds. Scale bars: 25 μm. Summary of the co-expression pattern between COX-1 and taste cell markers in the CV **(D)** and FP **(E)** of the mouse. The area of each ellipse indicates the number of taste bud cells expressing COX-1, T1R3, gustducin or CA4 in each taste papilla. Regions of overlap indicate co-expression. This diagram is based on Table 3 and previous study (Shigemura et al., 2008).

Next, we conducted double-immunostaining experiments to establish which taste bud cell types expressed COX-1 protein. A subset of COX-1-positive cells expressed T1R3, the sweet/umami receptor component (COX-1/T1R3: 54.4% in the CV, 60.6% in the FP; Figure 2A), and gustducin, the bitter/sweet/umami taste-related G protein (COX-1/gustducin: 40.6% in the CV; 48.0% in the FP; Figure 2B). However, only a few COX-1-positive cells expressed the sour taste cell marker, CA4 (COX-1/CA4: 7.8% in the CV, 9.8% in the FP; Figure 2C). COX-1-positive cells were also detected in the non-taste tissues around taste buds. Table 3 presents the inverse co-expression ratios (marker/COX-1), and a summary of the COX-1 and taste cell marker expression patterns is shown in Figure 2D (CV) and Figure 2E (FP). The positive signals were not detected when the primary antibody was omitted (Supplementary Figure 2). No significant differences in the number of positive cells per a taste bud were found between the sexes (Supplementary Figure 3).

**TABLE 3:** Co-expression ratios for prostaglandin-endoperoxide synthase 1 (Ptgs1)-positive taste cells and taste cell markers: gustducin (type II for bitter, sweet and umami), T1R3 (type II for sweet and umami) and carbonic anhydrase 4 (CA4, type III for sour) in mouse circumvallate (CV) and fungiform papillae (FP) in Figure 2.

Taste papillae		Percentage (%)		Percentage (%)		
CV	Ptgs1 (COX-1)/T1R3	54.4%	(403/741, *n* = 262)	T1R3/Ptgs1 (COX-1)	60.0%	(403/672, *n* = 262)
	Ptgs1 (COX-1)/Gustducin	40.6%	(189/465, *n* = 168)	Gustducin/Ptgs1 (COX-1)	52.6%	(189/359, *n* = 168)
	Ptgs1 (COX-1)/CA4	7.8%	(15/191, *n* = 119)	CA4/Ptgs1 (COX-1)	8.9%	(15/168, *n* = 119)
FP	Ptgs1 (COX-1)/T1R3	60.6%	(57/106, *n* = 33)	T1R3/Ptgs1 (COX-1)	53.8%	(57/94, *n* = 33)
	Ptgs1 (COX-1)/Gustducin	48.0%	(48/119, *n* = 37)	Gustducin/Ptgs1 (COX-1)	40.3%	(48/100, *n* = 37)
	Ptgs1 (COX-1)/CA4	9.8%	(9/99, *n* = 31)	CA4/Ptgs1 (COX-1)	9.1%	(9/92, *n* = 31)

(The number of protein A + B positive cells/that of protein B positive cells, *n* = the number of taste buds analyzed).

### 3.3 Cytosolic PGE synthase (cPGES) was also expressed mainly in type II taste cells

**FIGURE 3:**
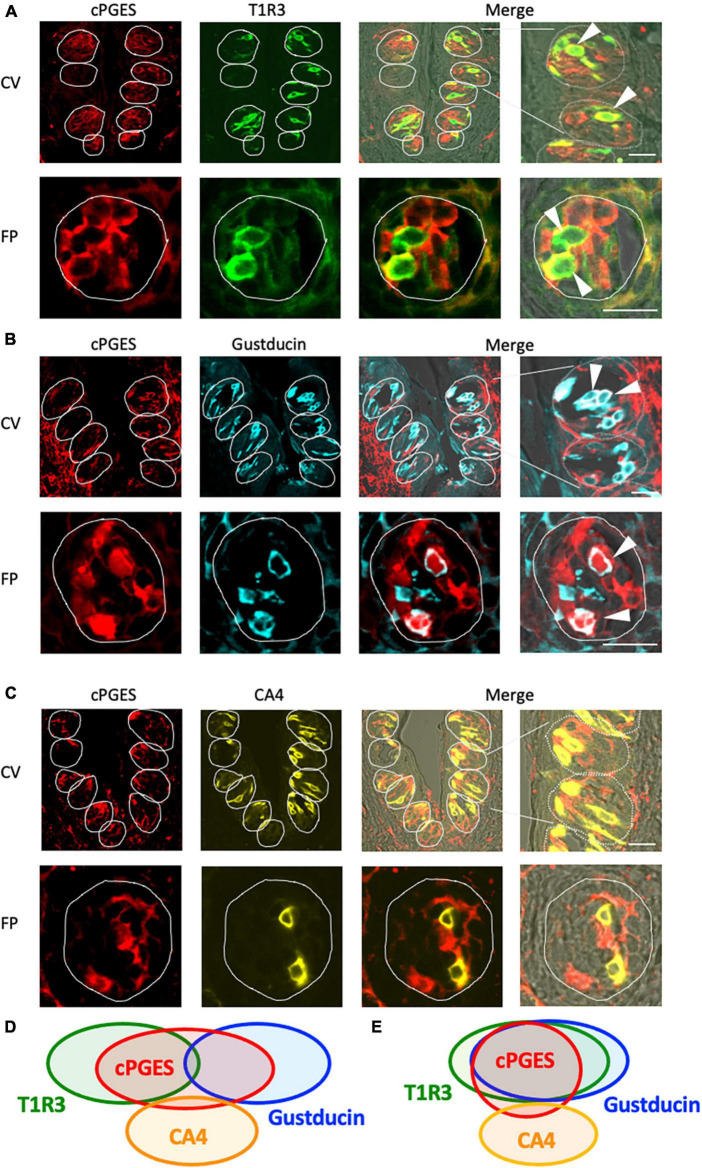
Cytosolic prostaglandin E synthase (cPGES) expression in the circumvallate papillae (CV) and fungiform papillae (FP) of mice. **(A,B)** cPGES immunoreactivity (red) in the CV and FP was observed mainly in taste bud type II cells. Arrowheads denote COX-1 + T1R3 (green) double-positive cells (yellow) **(A)**, and COX-1 + gustducin (magenta) double-positive cells (white) **(B)**. **(C)** A few cPGES-positive cells expressed the taste bud type III cell marker, carbonic anhydrase-4 (CA4, yellow). Dotted lines outline individual taste buds. Scale bars: 25 μm. Summary of the co-expression pattern between cPGES and taste cell markers in the CV **(D)** and FP **(E)** of the mouse. The area of each ellipse indicates the number of taste bud cells expressing cPGES, T1R3, gustducin or CA4 in each taste papilla. Regions of overlap indicate co-expression. This diagram is based on Table 4 and previous study (Shigemura et al., 2008).

The RT-PCR experiments described above confirmed the expression of *Pges* mRNA in mouse CV and FP. Molecular identification of multiple PGE synthase enzymes has revealed the existence of two segregated biosynthetic routes for PGE2 within the same cell, namely a constitutive COX-1-cPGES pathway and an inducible COX-2-membrane-associated PGE synthase pathway (Murakami et al., 2002). Therefore, we performed double-immunostaining experiments to determine which taste bud cell types expressed cPGES. We found that a subset of cPGES-positive cells expressed T1R3 (cPGES/T1R3: 43.1% in the CV, 60.6% in the FP; Figure 3A) and gustducin (cPGES/gustducin: 43.5% in the CV, 52.9% in the FP; Figure 3B), but very few cPGES-positive cells co-expressed CA4 (cPGES/CA4: 14.5% in the CV, 14.3% in the CV; Figure 3C). The inverse co-expression ratios (marker/cPGES) are shown in Table 4. cPGES-positive cells were also detected in the non-taste tissues around taste buds. A summary of the expression patterns for cPGES and taste cell markers is presented in Figure 3D (CV) and Figure 3E (FP). No significant differences in the number of positive cells per a taste bud were found between the sexes (Supplementary Figure 3).

**TABLE 4:** Co-expression ratios for cytosolic prostaglandin E synthase (cPGES)-positive taste cells and taste cell markers: gustducin (type II for bitter, sweet and umami), T1R3 (type II for sweet and umami) and carbonic anhydrase 4 (CA4, type III for sour) in mouse circumvallate (CV), and fungiform papillae (FP) in Figure 3.

Taste papillae		Percentage (%)		Percentage (%)		
CV	cPGES/T1R3	43.1%	(112/260, *n* = 111)	T1R3/cPGES	62.9%	(112/178, *n* = 111)
	cPGES/Gustducin	43.5%	(94/216, *n* = 80)	Gustducin/cPGES	55.6%	(94/169, *n* = 80)
	cPGES/CA4	14.5%	(19/131, *n* = 96)	CA4/cPGES	8.1%	(19/233, *n* = 96)
FP	cPGES/T1R3	60.6%	(28/53, *n* = 20)	T1R3/cPGES	49.1%	(28/57, *n* = 20)
	cPGES/Gustducin	52.9%	(27/51, *n* = 18)	Gustducin/cPGES	54.0%	(27/50, *n* = 18)
	cPGES/CA4	14.3%	(6/42, *n* = 19)	CA4/cPGES	12.2%	(6/49, *n* = 19)

(The number of protein A + B positive cells/that of protein B positive cells, *n* = the number of taste buds analyzed).

### 3.4 Diclofenac reduced the preference of mice for sweet and umami taste solutions but not for other basic taste solutions

**FIGURE 4:**
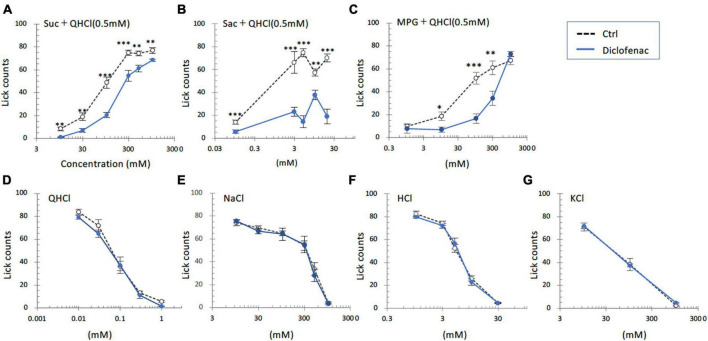
Diclofenac inhibited the behavioral responses of mice to sweet [sucrose (Suc), saccharin (Sac)] and umami [monopotassium glutamate (MPG)] substances. Lick counts are shown for varying concentrations of Suc combined with 0.5 mM quinine-HCl (QHCl) **(A)**, Sac combined with 0.5 mM QHCl **(B)**, MPG combined with 0.5 mM QHCl **(C)**, QHCl **(D)**, NaCl **(E)**, HCl **(F)**, and KCl **(G)**. The data were obtained after the intraperitoneal administration of vehicle (Ctrl, black dotted symbols) or 1.5 mg/kg body weight diclofenac (blue symbols) every day for 30 days. The lick counts are presented as the mean ± SEM (*n* = 4–18 mice). *P < 0.05, **P < 0.01, ***P < 0.001 (two-way ANOVA and *post-hoc* Student’s *t*-test; see Table 5).

In the subsequent series of experiments, we evaluated whether the administration of diclofenac for 30 days affected the behavioral responses of mice to various taste stimuli. The numbers of licks for sweet (Suc + QHCl or Sac + QHCl) and umami (MPG + QHCl) taste solution were significantly smaller for the diclofenac group than for the saline (control) group (*P* < 0.05, two-way ANOVA and *post-hoc* Student’s *t*-test; Figures 4A–C and Table 5). On the other hand, there were no significant differences between the diclofenac and saline groups in the lick counts for other taste solutions such as QHCl, NaCl, HCl, or KCl (*P* > 0.05, ANOVA; Figures 4D–G and Table 5).

**TABLE 5:** Results of statistical analysis for the effect of 30 days injection of diclofenac (Dic) in mice on the lick counts (Figure 4).

Figure	Content	Analysis			*P*-value
4A	Injection (Ctrl vs. Dic) × concentration (Sucrose + QHCl)	Two-way ANOVA	Injection (Ctrl vs. Dic)	*F*_(1, 112)_ = 58.494	<0.001
			Concentration	*F*_(5, 112)_ = 145.960	<0.001
			Injection × concentration	*F*_(5, 112)_ = 2.923	<0.05
		Student’s *t*-test	Ctrl vs. Dic	10 mM Suc + QHCl	<0.01
				30 mM Suc + QHCl	< 0.01
				100 mM Suc + QHCl	<0.001
				300 mM Suc + QHCl	<0.001
				500 mM Suc + QHCl	<0.01
				1000 mM Suc + QHCl	<0.01
4B	Injection (Ctrl vs. Dic) × concentration (Saccharin + QHCl)	Two-way ANOVA	Injection (Ctrl vs. Dic)	*F*_(1, 50)_ = 145.52	<0.001
			Concentration	*F*_(4, 50)_ = 22.263	<0.001
			Injection × concentration	*F*_(4, 50)_ = 10.427	<0.001
		Student’s *t*-test	Ctrl vs. Dic	0.1 mM Sac + QHCl	<0.001
				3 mM Sac + QHCl	<0001
				5 mM Sac + QHCl	<0.001
				10 mM Sac + QHCl	<0.01
				20 mM Sac + QHCl	<0.001
4C	Injection (Ctrl vs. Dic) × concentration (monopotassium glutamate + QHCl)	Two-way ANOVA	Injection (Ctrl vs. Dic)	*F*_(1, 103)_ = 22.479	<0.001
			Concentration	*F*_(4, 103)_ = 54.484	<0.001
			Injection × concentration	*F*_(4, 103)_ = 7.876	<0.001
		Student’s *t*-test	Ctrl vs. Dic	1 mM MPG + QHCl	0.658
				10 mM MPG + QHCl	<0.05
				100 mM MPG + QHCl	<0.001
				300 mM MPG + QHCl	<0.01
				1000 mM MPG + QHCl	0.229
4D	Injection (Ctrl vs. Dic) × concentration (QHCl)	Two-way ANOVA	Injection (Ctrl vs. Dic)	*F*_(1, 98)_ = 2.590	0.111
			Concentration	*F*_(4, 98)_ = 204.660	<0.001
			Injection × concentration	*F*_(4, 98)_ = 0.370	0.829
4E	Injection (Ctrl vs. Dic) × concentration (NaCl)	Two-way ANOVA	Injection (Ctrl vs. Dic)	*F*_(1, 48)_ = 0.907	0.346
			Concentration	*F*_(5, 48)_ = 325.331	<0.001
			Injection × concentration	*F*_(5, 48)_ = 0.806	0.551
4F	Injection (Ctrl vs. Dic) × concentration (HCl)	Two-way ANOVA	Injection (Ctrl vs. Dic)	*F*_(1, 40)_ = 0.113	0.739
			Concentration	*F*_(4, 40)_ = 340.375	<0.001
			Injection × concentration	*F*_(4, 40)_ = 0.687	0.605
4G	Injection (Ctrl vs. Dic) × concentration (KCl)	Two-way ANOVA	Injection (Ctrl vs. Dic)	*F*_(1, 32)_ = 0.060	0.808
			Concentration	*F*_(2, 32)_ = 89.140	<0.001
			Injection × concentration	*F*_(2, 32)_ = 0.065	0.937

### 3.5 Long-term or short-term administration of diclofenac reduced the CT nerve responses to sweet and umami taste solutions but not to other basic taste solutions

**FIGURE 5:**
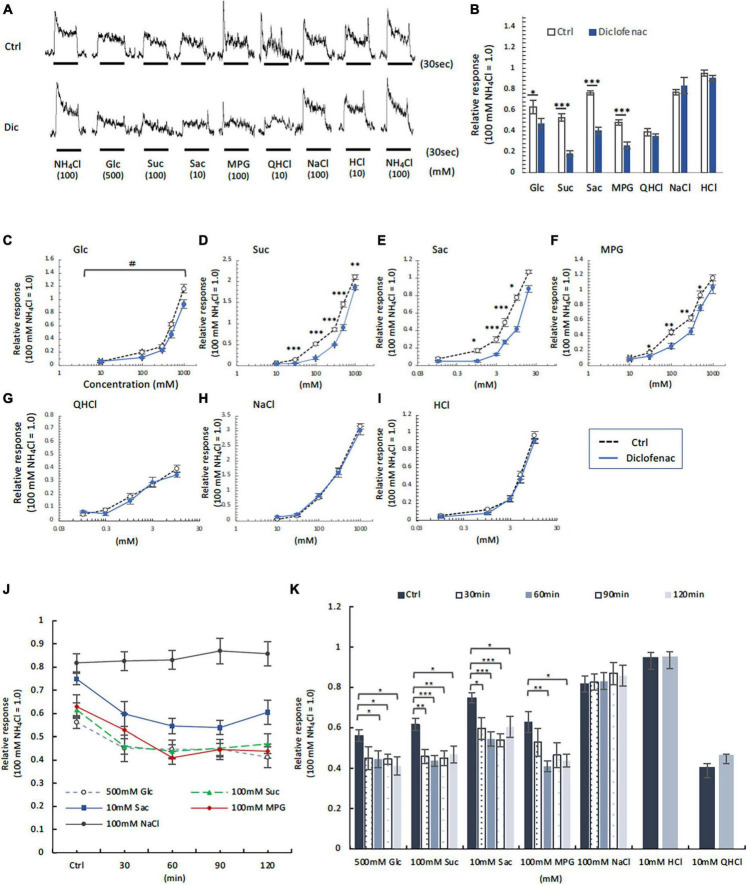
Diclofenac (Dic) affected the chorda tympani (CT) nerve responses to sweet [glucose (Glc), sucrose (Suc) and saccharin (Sac)] and umami [monopotassium glutamate (MPG)], but not to other basic taste stimuli. **(A–I)** Long-term administration of Dic. **(J,K)** A single administration of Dic. **(A)** Typical examples of CT nerve responses to 100 mM NH_4_Cl (NH_4_Cl), 500 mM Glc, 100 mM Suc, 10 mM Sac, 100 mM MPG, 10 mM quinine-HCl (QHCl), 100 mM NaCl, and 10 mM HCl. Bars indicate taste stimulation (30 s). **(B)** CT nerve responses to 500 mM Glc, 100 mM Suc, 10 mM Sac, 100 mM MPG, 10 mM QHCl, 100 mM NaCl, and 10 mM HCl after administration of vehicle (white bars, *n* = 6–15) or 1.5 mg/kg Dic (blue bars, *n* = 6–13) for 30 days. **(C–I)** Concentration-response relationships for the CT nerve responses to various tastants in mice administered vehicle (Ctrl, black symbols) or 1.5 mg/kg body weight Dic (blue symbols) every day for 30 days. The taste stimuli were 10–1000 mM Glc **(C)**, 10–1000 mM Suc **(D)**, 0.1–20 mM Sac **(E)**, 10–1000 mM MPG **(F)**, 0.1–10 mM QHCl **(G)**, 10–1000 mM NaCl **(H)**, and 0.1–10 mM HCl **(I)**. **(J,K)** Time-dependent changes in the CT nerve responses to 500 mM Glc (white circles), 100 mM Suc (green triangles), 10 mM Sac (blue squares), 100 mM MPG (red rhombi) and 100 mM NaCl (black circles) before (Ctrl) and 30–120 min after a single administration of 1.5 mg/kg Dic in mice (*n* = 5–7). The CT nerve responses are presented as the mean ± SEM. *P < 0.05, **P < 0.01, ***P < 0.001 (Ctrl vs. Dic; two-way ANOVA and *post-hoc* Student’s *t*-test; see Table 6), ^#^*P* < 0.001 [two-way ANOVA, injection (Ctrl vs. Dic); see Table 6].

Next, CT nerve recordings were used to assess the effects of short-term administration (a single dose) and long-term administration (once daily for 30 days) of diclofenac on taste responses in mice. The CT nerve responses to Glc (100, 500, and 1000 mM), Suc (30–1000 mM), Sac (1–10 mM) and MPG (30–500 mM) were significantly smaller in mice treated with diclofenac for 30 days than in control saline-treated mice (*P* < 0.05, two-way ANOVA and *post-hoc* Student’s *t*-test; Figures 5A–F and Table 6). On the other hand, there were no significant differences between groups in the responses to other taste qualities such as QHCl, NaCl and HCl (*P* > 0.05, ANOVA; Figures 5A, B, G–I and Table 6).

**TABLE 6:** Results of statistical analysis for the effect of 30 days or single injection of diclofenac (Dic) on the chorda tympani (CT) nerve responses in mice (Figure 5).

Figure	Content	Analysis			*P*-value
5B	Injection (Ctrl vs. Dic)	Student’s *t*-test	Ctrl vs. Dic	500 mM Glc	<0.05
				100 mM Suc	<0.001
				10 mM Sac	<0.001
				100 mM MPG	<0.001
				10 mM QHCl	0.397
				100 mM NaCl	0.462
				10 mM HCl	0.325
5C	Injection (Ctrl vs. Dic) × concentration (Glucose)	Two-way ANOVA	Injection (Ctrl vs. Dic)	*F*_(1, 80)_ = 15.531	<0.001
			Concentration	*F*_(4, 80)_ = 175.582	<0.001
			Injection × concentration	*F*_(4, 80)_ = 2.283	0.068
		Student’s *t*-test	Ctrl vs. Dic	10 mM Glc	0.896
				100 mM Glc	<0.05
				300 mM Glc	0.119
				500 mM Glc	<0.05
				1000 mM Glc	<0.05
5D	Injection (Ctrl vs. Dic) × concentration (Sucrose)	Two-way ANOVA	Injection (Ctrl vs. Dic)	*F*_(1, 114)_ = 158.893	<0.001
			Concentration	*F*_(5, 114)_ = 865.409	<0.001
			Injection × concentration	*F*_(5, 114)_ = 14.076	<0.001
		Student’s *t*-test	Ctrl vs. Dic	10 mM Suc	0.188
				30 mM Suc	<0.001
				100 mM Suc	<0.001
				300 mM Suc	<0.001
				500 mM Suc	<0.001
				1000 mM Suc	<0.01
5E	Injection (Ctrl vs. Dic) × concentration (Saccharin)	Two-way ANOVA	Injection (Ctrl vs. Dic)	*F*_(1, 86)_ = 113.909	<0.001
			Concentration	*F*_(5, 90)_ = 282.246	<0.001
			Injection × concentration	*F*_(5, 90)_ = 6.859	<0.001
		Student’s *t*-test	Ctrl vs. Dic	0.1 mM Sac	0.758
				1 mM Sac	<0.05
				3 mM Sac	<0.001
				5 mM Sac	<0.001
				10 mM Sac	<0.05
				20 mM Sac	0.281
5F	Injection (Ctrl vs. Dic) × concentration (Monopotassium glutamate)	Two-way ANOVA	Injection (Ctrl vs. Dic)	*F*_(1, 98)_ = 30.668	<0.001
			Concentration	*F*_(5, 98)_ = 222.853	<0.001
			Injection × concentration	*F*_(5, 98)_ = 1.998	<0.01
		Student’s *t*-test	Ctrl vs. Dic	10 mM MPG	0.458
				30 mM MPG	<0.05
				100 mM MPG	<0.01
				300 mM MPG	<0.01
				500 mM MPG	<0.05
				1000 mM MPG	0.207
5G	Injection (Ctrl vs. Dic) × concentration (QHCl)	Two-way ANOVA	Injection (Ctrl vs. Dic)	*F*_(1, 66)_ = 1.215	0.274
			Concentration	*F*_(4, 66)_ = 85.808	<0.001
			Injection × concentration	*F*_(4, 66)_ = 1.144	0.344
5H	Injection (Ctrl vs. Dic) × concentration (NaCl)	Two-way ANOVA	Injection (Ctrl vs. Dic)	*F*_(1, 67)_ = 0.018	0.893
			Concentration	*F*_(4, 67)_ = 436.43	<0.001
			Injection × concentration	*F*_(4, 67)_ = 0.630	0.643
5I	Injection (Ctrl vs. Dic) × concentration (HCl)	Two-way ANOVA	Injection (Ctrl vs. Dic)	*F*_(1, 71)_ = 0.024	0.076
			Concentration	*F*_(4, 71)_ = 63.015	<0.001
			Injection × concentration	*F*_(4, 71)_ = 0.330	0.914
5K	Injection (Ctrl vs. Dic)	Student’s *t*-test	500 mM Glc	30 min	0.091
				60 min	<0.05
				90 min	<0.05
				120 min	<0.05
			100 mM Suc	30 min	<0.01
				60 min	< 0.001
				90 min	<0.01
				120 min	<0.05
			10 mM Sac	30 min	<0.05
				60 min	<0.001
				90 min	<0.001
				120 min	<0.05
			100 mM MPG	30 min	0.265
				60 min	<0.01
				90 min	0.064
				120 min	<0.05
			100 mM NaCl	30 min	0.897
				60 min	0.826
				90 min	0.458
				120 min	0.552
			10 mM HCl		0.957
			10 mM QHCl		0.193

Chorda tympani nerve responses to various tastants (500 mM Glc, 100 mM Suc, 10 mM Sac, 100 mM MPG, 100 mM NaCl, 10 mM HCl and 10 mM QHCl) were also recorded at 0–120 min after the administration of a single dose of diclofenac. The CT nerve responses to sweet (Glc, Suc, and Sac) and umami (MPG) taste substances were significantly reduced at 60 min after diclofenac administration and remained below control levels at 120 min (*P* < 0.05, Student’s *t*-test; Figures 5J, K and Table 6). CT nerve responses to NaCl, HCl and QHCl were not affected by a single administration of diclofenac (*P* > 0.05, Student’s *t*-test; Figures 5J, K and Table 6). We also tested whether other NSAID, ibuprofen Na, affected the CT nerve responses to various tastants. As a result, similar to diclofenac, sweet and umami taste responses were significantly suppressed, and no effects were observed on the other taste qualities (Supplementary Figure 4).

### 3.6 Diclofenac decreased the mRNA expression levels of *Gnat3* (gustducin) and *cPges* in taste buds

**FIGURE 6:**
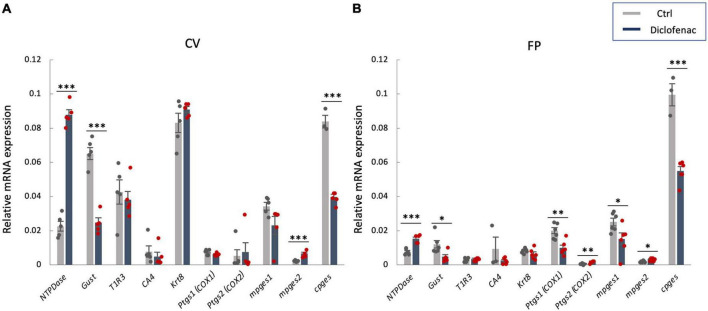
Real-time PCR was used to determine the mRNA expressions of taste-related genes in **(A)** the circumvallate papillae (CV) and **(B)** the fungiform papillae (FP) of mice administered vehicle (Ctrl, gray bars) or 1.5 mg/kg body weight diclofenac (Dic, blue bars) every day for 30 days. Prostaglandin-endoperoxide synthase 1/2 (*Ptgs1/Ptgs2*, *COX-1/-2*), microsomal/cytosolic prostaglandin E synthase (*m/cPges*), and taste cell markers: nucleoside triphosphate diphosphohydrolase (*NTPDase*, marker of type I cells), gustducin (*Gust*, marker of type II cells for bitter, sweet and umami tastes), *T1R3* (marker of type II cells for sweet and umami tastes), carbonic anhydrase-4 (*CA4*, marker of type III cells for sour taste), keratin-8 (*Krt8*, marker of differentiated taste cells). Data were obtained from at least three independent experiments per group, and each PCR assay was performed in duplicate. The quantitative PCR results were normalized using the ΔΔCt method with glyceraldehyde-3-phosphate dehydrogenase (*Gapdh*) as the reference. All data are presented as the mean ± SEM (Ctrl, *n* = 3–6 mice; Dic, *n* = 3–6 mice). *P < 0.05, **P < 0.01, ***P < 0.001 (Student’s *t*-test; see Table 7).

In order to investigate the molecular mechanisms underlying the reduction of sweet/umami taste sensitivity after the administration of diclofenac for 30 days, we used q-PCR to examine the mRNA expression levels of diclofenac’s targets, *Ptgs1* (*COX-1*) and *Ptgs2* (*COX-2*), and downstream molecules, membrane-associated prostaglandin E synthases (*mPges1* and *mPges2*) and cPGES (*cPges*), as well as taste cell markers in taste bud cells. The results showed that the relative expression levels of *Gnat3* mRNA (gustducin, a bitter/sweet/umami-related G protein) and *cPges* mRNA in both the CV and FP were significantly lower in diclofenac-treated mice than in vehicle-treated mice (*P* < 0.05, Student’s *t*-test; Figures 6A, B and Table 7). Furthermore, the mRNA expression levels of *Entpd2* (NTPDase-2) and *mPges2* (which was expressed at a low level) were increased in both the CV and FP in diclofenac-treated mice compared to vehicle-treated mice (*P* < 0.05, Student’s *t*-test; Figures 6A, B and Table 7). Diclofenac also decreased the expression levels of *Ptgs1* and *mPges1* mRNA and increased the level of *Ptgs2* mRNA (which was expressed at a low level) in the FP (*P* < 0.05, Student’s *t*-test; Figures 6A, B and Table 7). The mRNA levels for the other taste cell markers, T1R3 (*Tas1r3*) and CA4 (*Ca4*), and for the differentiated taste cell marker, keratin-8 (*Krt8*), were not altered by the administration of diclofenac (*P* > 0.05, Student’s *t*-test; Figures 6A, B and Table 7).

**TABLE 7:** Results of statistical analysis for the effect of injection of diclofenac (Dic) on the gene expression in the circumvallate papillae (CV) and fungiform papillae (FP) of mice by real-time PCR (Figure 6).

Figure	Taste papillae	Analysis			*P*-value
6A	CV	Student’s *t*-test	Ctrl vs. Dic	*NTPDase*	<0.001
				*Gustducin*	<0.001
				*T1R3*	0.616
				*CA4*	0.506
				*Krt8*	0.220
				*Ptgs1*	0.119
				*Ptgs2*	0.745
				*mPges1*	0.086
				*mPges2*	<0.001
				*cPges*	<0.001
6B	FP	Student’s *t*-test	Ctrl vs. Dic	*NTPDase*	<0.001
				*Gustducin*	<0.05
				*T1R3*	0.394
				*CA4*	0.190
				*Krt8*	0.127
				*Ptgs1*	<0.01
				*Ptgs2*	<0.01
				*mPges1*	<0.05
				*mPges2*	<0.05
				*cPges*	<0.001

### 3.7 Ca^2+^ imaging

**FIGURE 7:**
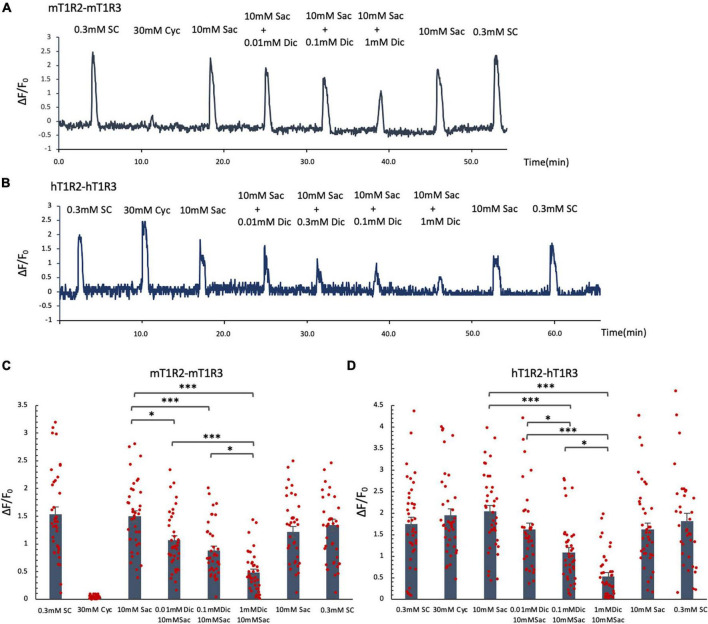
Diclofenac (Dic) inhibited the activation of mouse and human T1R2/T1R3 sweet taste receptors. Human and mouse sweet receptor heterodimers (hT1R2/hT1R3 and mT1R2/mT1R3, respectively) were expressed in HEK293 cells along with a Gα subunit (G16-gust44). Calcium mobilization was measured in response to each of the following sweeteners: 0.3 mM SC45647 (SC), 30 mM cyclamate (Cyc), 10 mM saccharin (Sac), and 10 mM Sac mixed with Dic (0.01 mM, 0.1 mM, 1 mM). **(A,B)** Typical examples of calcium mobilization mediated by mouse and human T1R2/T1R3 receptors. **(C,D)** Data are expressed as the mean ± SEM of *n* = 38 cells (three independent experiments per group). *P < 0.05, ***P < 0.001 [one-way ANOVA among 10 mM Sac and 10 mM Sac + Dic (0.01 mM, 0.1 mM, 1 mM) and followed by *post-hoc* Tukey’s *t*-test; see Table 8].

Since the administration of a single dose of diclofenac was observed to reduce the sensitivity to sweet and umami tastes (Figures 5J, K), we tested whether diclofenac inhibited the activity of the T1R2/T1R3 sweet taste receptor using Ca^2+^ imaging and a HEK293 cell heterologous expression system. We found that diclofenac caused a concentration-dependent inhibition of the activation of the mouse sweet taste receptor (mT1R2/mT1R3) in HEK293 cells (*P* < 0.05, one-way ANOVA and *post-hoc* Tukey’s test; Figure 7 and Table 8). A similar concentration-dependent inhibition of activation was observed for the human sweet taste receptor (hT1R2/hT1R3) in HEK293 cells (*P* < 0.05, one-way ANOVA and *post-hoc* Tukey’s test; Figure 7 and Table 8). Diclofenac did not inhibit the activation of the endogenous beta-adrenergic receptor (belonging to the large family of G protein-coupled receptors, GPCRs) by application of isoproterenol in HEK293 cells (Supplementary Figure 5).

**TABLE 8:** Results of statistical analysis for the effect of addition of diclofenac (Dic) on the Ca imaging in human and mouse sweet receptor heterodimers (hT1R2/hT1R3, mT1R2/mT1R3; Figure 7).

Figure	Content	Analysis			*P*-value
7C	mT1R2-mT1R3	One-way ANOVA	*F*_(3,__148)_ = 26.321		<0.001
		*Post-hoc* Türkiye test	Ctrl vs. Dic	10 mM Sac vs. 10 mM Sac + 0.01 mM Dic	0.05
				10 mM Sac vs. 10 mM Sac + 0.1 mM Dic	<0.001
				10 mM Sac vs. 10 mM Sac + 1 mM Dic	<0.001
				10 mM Sac + 0.01 mM Dic vs. 10 mM Sac + 0.1 mM Dic	0.377
				10 mM Sac + 0.01 mM Dic vs. 10 mM Sac + 1 mM Dic	<0.001
				10 mM Sac + 0.1 mM Dic vs. 10 mM Sac + 1 mM Dic	<0.05
7D	hT1R2-hT1R3	One-way ANOVA	*F*_(3,__151)_ = 27.717		<0.001
		*Post-hoc* Türkiye test	Ctrl vs. Dic	10 mM Sac vs. 10 mM Sac + 0.01 mM Dic	0.085
				10 mM Sac vs. 10 mM Sac + 0.1 mM Dic	<0.001
				10 mM Sac vs. 10 mM Sac + 1 mM Dic	<0.001
				10 mM Sac + 0.01 mM Dic vs. 10 mM Sac + 0.1 mM Dic	<0.05
				10 mM Sac + 0.01 mM Dic vs. 10 mM Sac + 1 mM Dic	<0.001
				10 mM Sac + 0.1 mM Dic vs. 10 mM Sac + 1 mM Dic	<0.05

## 4 Discussion

The main aim of this study was to provide insights into the molecular mechanisms underlying diclofenac-induced taste disorders. Several clinical reports have described taste disorders associated with NSAIDs. For example, patients taking diclofenac sodium were found to have decreased taste sensitivity to sucrose and citric acid (Schiffman et al., 2000). Furthermore, taste disorders were observed in 4.2% of patients taking an oral NSAID (celecoxib) (Lipton et al., 2020) and 9.9% of patients (mild taste disturbance, 2.0%; moderate, 5.9%; severe, 2.0%) administered an intranasal NSAID (Ketorolac) (Rao et al., 2016). Therefore, we speculated that diclofenac-induced taste disturbance might occur due to inhibition of COX (the target of NSAIDs) and downstream effects on the arachidonic acid pathway in taste bud cells. The COX-1 isoform is constitutively present in various cell types, whereas COX-2 is inducible and normally absent from cells (Vane et al., 1998). Previous single cell RNA sequencing analyses revealed that *Ptgs1* mRNA (COX-1) was expressed abundantly in mouse T1R3-positive taste cells but not in mouse type III sour-responsive taste cells, while *Ptgs2* mRNA (COX-2) was barely detected in either cell type (Sukumaran et al., 2017). In view of this, the present study focused on the constitutively active isoform, COX-1, rather than on COX-2. We found that COX-1 and cPGES were expressed in a subset of mouse taste bud cells in the CV and FP that also expressed T1R3 (a sweet/umami taste receptor component) and/or gustducin (a bitter/sweet/umami taste-related G protein). Whereas, COX-1 and cPGES were rarely expressed in other types of taste cell (CA4-positive cells mediating sour taste) in the taste buds. COX-1 and cPGES ware also expressed in non-taste tongue epithelial cells around taste buds (Figures 1–3). Both the behavioral and neural analyses revealed that mice administered diclofenac for 30 days exhibited significantly reduced responses to sweet and umami taste solutions but not to other tastants such as NaCl, QHCl or HCl (Figures 4, 5). These results suggest that diclofenac inhibits the neural and behavioral responses to sweet and umami tastants by directly acting on mainly peripheral T1R3-positive and/or gustducin-positive sweet/umami taste cells that express COX-1 and cPGES.

The arachidonic acid pathway plays important roles in cardiovascular biology, carcinogenesis and many inflammatory diseases such as asthma and arthritis (Wang et al., 2021). Esterified arachidonic acid on the inner surface of the cell membrane is hydrolyzed by PLA2 and further metabolized by COX isoforms to produce PGH2, which is metabolized by PG synthases to a spectrum of bioactive mediators (PGE2, PGI2, PGD2, PGF2α, and TXA2). It has been reported that COX-1 and COX-2 generate PGs in the stomach and intestine to help to maintain the integrity of the mucosal epithelium (Kargman et al., 1996). Patients with rheumatoid arthritis exhibit small intestinal ulceration after long-term NSAID use (Kent et al., 1969; Morris et al., 1991), and the prevalence of non-specific ulceration is slightly higher for long-term NSAID use (prescribed daily for at least 6 months) than for short-term NSAID use (prescribed daily for less than 6 months or discontinuous courses prescribed for 6 months) (Allison et al., 1992). Radiation injury increases COX-1 levels in intestinal crypt stem cells, and PGE2 produced through COX-1 is thought to promote crypt stem cell survival and proliferation (Cohn et al., 1997). Additionally, gastric mucosal injury induced by 0.6 N HCl was more severe in COX-1 knockout mice than in wild-type mice or COX-2 knockout mice (Darling et al., 2004). Furthermore, it has been suggested that there may be COX/prostaglandin pathway in murine intestinal brush (tuft) cells expressing TRPM5 (Bezençon et al., 2008). In the present study, almost all the arachidonic acid pathway genes were detected in mouse taste buds. Furthermore, long-term administration of diclofenac suppressed the mRNA expression of *Ptgs-1* (COX-1), *Pges* (PGE synthase), and *Gnat3* (gustducin, a bitter/sweet/umami-related G protein) in taste buds of both the mouse CV and FP. Long-term diclofenac administration also increased the expression level of *Entpd2* (NTPDase-2, a type I taste cell marker) but was not associated with any changes in the mRNA levels of *Ca4* (a type III taste cell marker) or *Krt8* (keratin-8, a differentiated taste cell marker) (Figure 6). These results suggest that the COX/PG synthase pathway may mediate constitutive PG synthesis in sweet/umami taste cells, and it is possible that this may play an important role in maintaining the integrity of these cells, as is the case for gut mucosal cells. Long-term diclofenac administration may disturb the COX/PG synthase pathway and cause a reduction in sweet/umami-responsive components in the taste buds, thereby specifically decreasing the neural and behavioral responses to sweet/umami stimuli.

Interestingly, the present study showed that a single application of diclofenac also suppressed the mouse CT nerve responses to sweet/umami substances such as Glc, Suc, Sac, and MPG but not to other taste stimuli (Figures 5J, K). Diclofenac inhibited the activation of not only the mouse sweet taste receptor (mT1R2/mT1R3) but also the human sweet taste receptor (hT1R2/hT1R3) in a HEK293 cell heterologous expression system (Figure 7). Diclofenac did not inhibit the activation of the beta-adrenergic receptor (same GPCR as sweet taste receptor) by application of isoproterenol in HEK293 cells (Supplementary Figure 5), suggesting that the inhibitory effect of diclofenac acts specifically on the sweet taste receptors, and is not a non-specific effect on Ca^2+^ dynamics in HEK293 cells. A previous study investigated the effect of ibuprofen (a NSAID) on the activity of the sweet taste receptor, given the similarity in structure (α-methylacetic acid) between ibuprofen and a sweet taste inhibitor, lactisole. Ibuprofen was found to be a potent inhibitor of the human T1R2/T1R3 sweet taste receptor, and docking simulations suggested that ibuprofen bound to the transmembrane region of T1R3 to exert an inhibitory effect similar to that of lactisole (Nakagita et al., 2020). Based on chemical structure, diclofenac is classified as a 2-arylacetic acid derivative whereas ibuprofen is classified as a propionic acid derivative, but both are weak organic acids comprising an acidic moiety and an aromatic functional group. At a concentration that inhibits 80% of COX-2 activity, diclofenac inhibits about 70% of COX-1 activity, while ibuprofen inhibits about 85% of COX-1 activity (Warner et al., 1999). Furthermore, both diclofenac and ibuprofen are classified as reversible COX inhibitors in terms of kinetics (Vonkeman and van de Laar, 2010), and both drugs are short-acting NSAIDs metabolized by cytochrome 2c9 (plasma half-life less than 6 h) (Conaghan, 2012; Bindu et al., 2020). Thus, diclofenac and ibuprofen have relatively similar structures, COX-selective inhibitory actions and kinetics, raising the possibility that diclofenac might act not only on the COX-1/cPGES pathway in taste bud cells but also directly on the T1R2/T1R3 sweet taste receptor in mice. It is possible that diclofenac exerts a suppressive effect on the T1R2/T1R3 receptor via an interaction with the transmembrane region of T1R3 (a component common to both sweet and umami receptors), similar to that of lactisole and ibuprofen, and this may explain why diclofenac affected both sweet and umami taste responses in mice. In this study, a single administration of ibuprofen also specifically suppressed the mouse chorda tympani nerve responses to sweet and umami substances, but not to the other basic taste stimuli (Supplementary Figure 4). This may support the above hypothesis, and suggests that NSAIDs with structural similarity to diclofenac may cause taste disorders through the common mechanism of action. Thus, the diclofenac-induced specific reduction of sweet and umami taste sensitivity may be mediated by both direct effects on the T1R2/T1R3 sweet taste receptor (acute phase: reversible suppression) and indirect effects on the mRNA expression of sweet/umami responsive components via COX-1/cPGES pathway in taste bud cells (chronic phase: persistent suppression). Such a cumulative suppression of peripheral taste sensitivity may underlie the diclofenac-induced taste alterations. Although the expression level of gustducin (bitter/sweet/umami) mRNA was significantly reduced by the application of diclofenac for 30 days, the bitter taste response was not significantly reduced. This may be because the direct effect on T1R2/T1R3 is faster and more effective than slow and cumulative effects on COX-1/cPGES pathway, which makes it difficult to see changes in bitter taste responses.

As mentioned above, PGs synthesized by COX are thought to play an important role in the maintenance of gut mucosal integrity. Thus, gastrointestinal adverse events are the most frequent side effects reported for NSAIDs (García Rodríguez and Jick, 1994). In general, ibuprofen and diclofenac have the lowest risk of adverse events among early NSAIDs, especially when administered at low doses (<75 mg daily) (Langman et al., 1994). In this study, the dose of diclofenac administered to the mice (1.5 mg/kg body weight) was determined with reference to the dose for humans stated in the prescribing information (75–100 mg per day, equivalent to 1.25–1.7 mg/kg body weight). It should be noted that there was no significant difference in body weight between diclofenac-treated and control (saline-treated) mice (Supplementary Figure 6). Furthermore, there were also no significant differences in lick numbers for NaCl, QHCl or HCl between these two groups. These findings indicate that the administration of diclofenac at the concentrations used in this study do not cause severe gastrointestinal injury or affect appetite, cognition or drinking in mice.

Diclofenac sodium solution has a primarily bitter taste when presented to the human tongue, with additional medicinal, metallic, astringent and salty characteristics. Lingual application of diclofenac also suppresses the taste responses to sucrose, citric acid and capsaicin, and detection thresholds for young subjects were found to be around 1 mM (Schiffman et al., 2000). Therefore, we administered diclofenac intraperitoneally rather than orally in this study. The expected maximum plasma concentration of diclofenac after intraperitoneal administration in mice is approximately 0.005 mM, which is far below the detection threshold observed in the human study (1 mM); hence, even if diclofenac was present in the saliva at this concentration, it would not be expected to affect taste responses as a bitter stimulant.

In conclusion, the findings of this study indicate that mouse taste tissues contain a functional COX-1/prostaglandin synthase pathway. Long-term administration of the COX inhibitor, diclofenac, inhibited the neural and behavioral responses to sweet and umami substances and reduced the mRNA expression of *Ptgs-1* (COX-1), *cPges* (PGE synthase) and *Gnat3* (gustducin) in mouse taste buds. Furthermore, diclofenac inhibited the activation of both mouse and human T1R2/T1R3 sweet taste receptors expressed in HEK293 cells. These results suggest that diclofenac may suppress the activation of sweet and umami taste cells acutely via a direct action on T1R3 (which is a component of both sweet and umami taste receptors) and chronically via inhibition of the COX/prostaglandin synthase pathway, which leads to a reduction in the mRNA expression of sweet/umami responsive components. This dual inhibition mechanism may underlie diclofenac-induced taste alterations in humans. These findings provide new insights into the molecular mechanisms of drug-induced taste disorders. Tongue icing to prevent diclofenac from reaching the taste buds through the bloodstream, and/or applying cyclamate-like sweet enhancing compounds which competitively bind to the T1R3 transmembrane region, may partially improve the quality of life for patients relying on this medication for pain and inflammation management.

## Data availability statement

The original contributions presented in the study are included in the article/Supplementary material, further inquiries can be directed to the corresponding author.

## Ethics statement

Ethical approval was not required for the studies on humans in accordance with the local legislation and institutional requirements because only commercially available established cell lines were used. The animal study was approved by National Institutes of Health Guide for the Care and Use of Laboratory Animals and approved by the committee for Laboratory Animal Care and Use at Kyushu University, Japan (approval no. A21-002-0, 14 September 2020). The study was conducted in accordance with the local legislation and institutional requirements.

## Author contributions

NS: Conceptualization, Data curation, Funding acquisition, Methodology, Project administration, Supervision, Writing – original draft, Writing – review and editing. AH: Conceptualization, Data curation, Funding acquisition, Investigation, Methodology, Validation, Visualization, Writing – original draft, Writing – review and editing. SI: Data curation, Investigation, Methodology, Supervision, Validation, Visualization, Writing – review and editing. AO: Investigation, Methodology, Validation, Visualization, Writing – review and editing. YK: Investigation, Validation, Writing – review and editing. YN: Investigation, Writing – review and editing. ST: Investigation, Methodology, Validation, Visualization, Writing – review and editing. KS: Investigation, Methodology, Validation, Visualization, Writing – review and editing. IT: Project administration, Supervision, Validation, Writing – review and editing.

## References

[B1] Allison M. C. Howatson A. G. Torrance C. J. Lee F. D. Russell R. I. (1992). Gastrointestinal damage associated with the use of nonsteroidal antiinflammatory drugs. *N. Engl. J. Med.* 327 749–754. 10.1056/NEJM199209103271101 1501650

[B2] Beidler L. M. Smallman R. L. (1965). Renewal of cells within taste buds. *J. Cell Biol.* 27 263–272. 10.1083/jcb.27.2.263 5884625 PMC2106718

[B3] Bezençon C. Fürholz A. Raymond F. Mansourian R. Métairon S. Le Coutre J. (2008). Murine intestinal cells expressing Trpm5 are mostly brush cells and express markers of neuronal and inflammatory cells. *J. Comp. Neurol.* 509 514–525. 10.1002/cne.21768 18537122

[B4] Bindu S. Mazumder S. Bandyopadhyay U. (2020). Non-steroidal anti-inflammatory drugs (NSAIDs) and organ damage: A current perspective. *Biochem. Pharmacol.* 180:114147. 10.1016/j.bcp.2020.114147 32653589 PMC7347500

[B5] Chandrashekar J. Hoon M. A. Ryba N. J. Zuker C. S. (2006). The receptors and cells for mammalian taste. *Nature* 444 288–294. 10.1038/nature05401 17108952

[B6] Chandrashekar J. Yarmolinsky D. von Buchholtz L. Oka Y. Sly W. Ryba N. J. (2009). The taste of carbonation. *Science* 326 443–445. 10.1126/science.1174601 19833970 PMC3654389

[B7] Chaudhari N. Roper S. D. (2010). The cell biology of taste. *J. Cell Biol.* 190 285–296. 10.1083/jcb.201003144 20696704 PMC2922655

[B8] Cohn S. M. Schloemann S. Tessner T. Seibert K. Stenson W. F. (1997). Crypt stem cell survival in the mouse intestinal epithelium is regulated by prostaglandins synthesized through cyclooxygenase-1. *J. Clin. Invest.* 99 1367–1379. 10.1172/JCI119296 9077547 PMC507953

[B9] Conaghan P. G. (2012). A turbulent decade for NSAIDs: Update on current concepts of classification, epidemiology, comparative efficacy, and toxicity. *Rheumatol. Int.* 32 1491–1502. 10.1007/s00296-011-2263-6 22193214 PMC3364420

[B10] Darling R. L. Romero J. J. Dial E. J. Akunda J. K. Langenbach R. Lichtenberger L. M. (2004). The effects of aspirin on gastric mucosal integrity, surface hydrophobicity, and prostaglandin metabolism in cyclooxygenase knockout mice. *Gastroenterology* 127 94–104. 10.1053/j.gastro.2004.04.003 15236176

[B11] Farbman A. I. (1980). Renewal of taste bud cells in rat circumvallate papillae. *Cell Tissue Kinet* 13 349–357. 10.1111/j.1365-2184.1980.tb00474.x 7428010

[B12] Gan T. J. (2010). Diclofenac: An update on its mechanism of action and safety profile. *Curr. Med. Res. Opin.* 26 1715–1731. 10.1185/03007995.2010.486301 20470236

[B13] García Rodríguez L. A. Jick H. (1994). Risk of upper gastrointestinal bleeding and perforation associated with individual non-steroidal anti-inflammatory drugs. *Lancet* 343 769–772. 10.1016/s0140-6736(94)91843-0 7907735

[B14] Grosser T. Fries S. FitzGerald G. A. (2006). Biological basis for the cardiovascular consequences of COX-2 inhibition: Therapeutic challenges and opportunities. *J. Clin. Invest.* 116 4–15. 10.1172/JCI27291 16395396 PMC1323269

[B15] He W. Yasumatsu K. Varadarajan V. Yamada A. Lem J. Ninomiya Y. (2004). Umami taste responses are mediated by alpha-transducin and alpha-gustducin. *J. Neurosci.* 24 7674–7680. 10.1523/JNEUROSCI.2441-04.2004 15342734 PMC6729622

[B16] Kargman S. Charleson S. Cartwright M. Frank J. Riendeau D. Mancini J. (1996). Characterization of Prostaglandin G/H Synthase 1 and 2 in rat, dog, monkey, and human gastrointestinal tracts. *Gastroenterology* 111 445–454. 10.1053/gast.1996.v111.pm8690211 8690211

[B17] Kent T. H. Cardelli R. M. Stamler F. W. (1969). Small intestinal ulcers and intestinal flora in rats given indomethacin. *Am. J. Pathol.* 54 237–249. 5765565 PMC2013470

[B18] Ku E. C. Lee W. Kothari H. V. Scholer D. W. (1986). Effect of diclofenac sodium on the arachidonic acid cascade. *Am. J. Med.* 80 18–23. 10.1016/0002-9343(86)90074-4 3085488

[B19] Langman M. J. Weil J. Wainwright P. Lawson D. H. Rawlins M. D. Logan R. F. (1994). Risks of bleeding peptic ulcer associated with individual non-steroidal anti-inflammatory drugs. *Lancet* 343 1075–1078. 10.1016/s0140-6736(94)90185-6 7909103

[B20] Lindemann B. (2001). Receptors and transduction in taste. *Nature* 413 219–225. 10.1038/35093032 11557991

[B21] Lipton R. B. Munjal S. Brand-Schieber E. Tepper S. J. Dodick D. W. (2020). Efficacy, tolerability, and safety of DFN-15 (Celecoxib oral solution, 25 mg/mL) in the acute treatment of episodic migraine: A randomized, double-blind, placebo-controlled study. *Headache* 60 58–70. 10.1111/head.13663 31647577 PMC7003821

[B22] Morris A. J. Madhok R. Sturrock R. D. Capell H. A. MacKenzie J. F. (1991). Enteroscopic diagnosis of small bowel ulceration in patients receiving non-steroidal anti-inflammatory drugs. *Lancet* 337:520. 10.1016/0140-6736(91)91300-j 1671893

[B23] Murakami M. Nakatani Y. Tanioka T. Kudo I. (2002). Prostaglandin E synthase. *Prostaglandins Other Lipid Mediat.* 6 383–399. 10.1016/s0090-6980(02)00043-6 12432931

[B24] Murata Y. Nakashima K. Yamada A. Shigemura N. Sasamoto K. Ninomiya Y. (2003). Gurmarin suppression of licking responses to sweetener-quinine mixtures in C57BL mice. *Chem. Senses* 28 237–243. 10.1093/chemse/28.3.237 12714446

[B25] Nakagita T. Taketani C. Narukawa M. Hirokawa T. Kobayashi T. Misaka T. (2020). Ibuprofen, a nonsteroidal anti-inflammatory drug, is a potent inhibitor of the human sweet taste receptor. *Chem. Senses* 45 667–673. 10.1093/chemse/bjaa057 32832995

[B26] Oike A. Iwata S. Hirayama A. Ono Y. Nagasato Y. Kawabata Y. (2022). Bisphosphonate affects the behavioral responses to HCl by disrupting farnesyl diphosphate synthase in mouse taste bud and tongue epithelial cells. *Sci. Rep.* 12:21246. 10.1038/s41598-022-25755-5 36481783 PMC9732047

[B27] Oike H. Wakamori M. Mori Y. Nakanishi H. Taguchi R. Misaka T. (2006). Arachidonic acid can function as a signaling modulator by activating the TRPM5 cation channel in taste receptor cells. *Biochim. Biophys. Acta* 1761 1078–1084. 10.1016/j.bbalip.2006.07.005 16935556

[B28] Patrono C. Patrignani P. García Rodríguez L. A. (2001). Cyclooxygenase-selective inhibition of prostanoid formation: Transducing biochemical selectivity into clinical read-outs. *J. Clin. Invest.* 108 7–13. 10.1172/JCI13418 11435450 PMC209347

[B29] Rao A. S. Gelaye B. Kurth T. Dash P. D. Nitchie H. Peterlin B. L. (2016). A randomized trial of ketorolac vs. sumatripan vs. Placebo Nasal Spray (KSPN) for acute migraine. *Headache.* 56 331–340. 10.1111/head.12767 26840902 PMC4822712

[B30] Sanematsu K. Kitagawa M. Yoshida R. Nirasawa S. Shigemura N. Ninomiya Y. (2016). Intracellular acidification is required for full activation of the sweet taste receptor by miraculin. *Sci. Rep.* 6:22807. 10.1038/srep22807 26960429 PMC4785348

[B31] Sanematsu K. Kusakabe Y. Shigemura N. Hirokawa T. Nakamura S. Imoto T. (2014). Molecular mechanisms for sweet-suppressing effect of gymnemic acids. *J. Biol. Chem.* 289 25711–25720. 10.1074/jbc.M114.560409 25056955 PMC4162174

[B32] Sanematsu K. Yamamoto M. Nagasato Y. Kawabata Y. Watanabe Y. Iwata S. (2023). Prediction of dynamic allostery for the transmembrane domain of the sweet taste receptor subunit, TAS1R3. *Commun. Biol.* 6:340. 10.1038/s42003-023-04705-5 37012338 PMC10070457

[B33] Schiffman S. S. Zervakis J. Westall H. L. Graham B. G. Metz A. Bennett J. L. (2000). Effect of antimicrobial and anti-inflammatory medications on the sense of taste. *Physiol. Behav.* 69 413–424. 10.1016/s0031-9384(99)00262-0 10913779

[B34] Shigemura N. Ninomiya Y. (2016). Recent advances in molecular mechanisms of taste signaling and modifying. *Int. Rev. Cell Mol. Biol.* 323 71–106. 10.1016/bs.ircmb.2015.12.004 26944619

[B35] Shigemura N. Iwata S. Yasumatsu K. Ohkuri T. Horio N. Sanematsu K. (2013). Angiotensin II modulates salty and sweet taste sensitivities. *J. Neurosci.* 33 6267–6277. 10.1523/JNEUROSCI.5599-12.2013 23575826 PMC6619077

[B36] Shigemura N. Nakao K. Yasuo T. Murata Y. Yasumatsu K. Nakashima A. (2008). Gurmarin sensitivity of sweet taste responses is associated with co-expression patterns of T1r2, T1r3, and gustducin. *Biochem. Biophys. Res. Commun.* 367 356–363. 10.1016/j.bbrc.2007.12.146 18174025

[B37] Shigemura N. Ohta R. Kusakabe Y. Miura H. Hino A. Koyano K. (2004). Leptin modulates behavioral responses to sweet substances by influencing peripheral taste structures. *Endocrinology* 145 839–847. 10.1210/en.2003-0602 14592964

[B38] Sukumaran S. K. Lewandowski B. C. Qin Y. Kotha R. Bachmanov A. A. Margolskee R. F. (2017). Whole transcriptome profiling of taste bud cells. *Sci. Rep.* 7:7595. 10.1038/s41598-017-07746-z 28790351 PMC5548921

[B39] Takai S. Yasumatsu K. Inoue M. Iwata S. Yoshida R. Shigemura N. (2015). Glucagon-like peptide-1 is specifically involved in sweet taste transmission. *FASEB J.* 29 2268–2280. 10.1096/fj.14-265355 25678625 PMC4763871

[B40] Tu Y. H. Cooper A. J. Teng B. Chang R. B. Artiga D. J. Turner H. N. (2018). An evolutionarily conserved gene family encodes proton-selective ion channels. *Science* 359 1047–1050. 10.1126/science.aao3264 29371428 PMC5845439

[B41] Vane J. R. Bakhle Y. S. Botting R. M. (1998). Cyclooxygenases 1 and 2. *Annu. Rev. Pharmacol. Toxicol.* 38 97–120. 10.1146/annurev.pharmtox.38.1.97 9597150

[B42] Vonkeman H. E. van de Laar M. A. (2010). Nonsteroidal anti-inflammatory drugs: Adverse effects and their prevention. *Semin. Arth. Rheum.* 39 294–312. 10.1016/j.semarthrit.2008.08.001 18823646

[B43] Wang B. Wu L. Chen J. Dong L. Chen C. Wen Z. (2021). Metabolism pathways of arachidonic acids: Mechanisms and potential therapeutic targets. *Signal. Transduct Target Ther.* 6:94. 10.1038/s41392-020-00443-w 33637672 PMC7910446

[B44] Warner T. D. Giuliano F. Vojnovic I. Bukasa A. Mitchell J. A. Vane J. R. (1999). Nonsteroid drug selectivities for cyclo-oxygenase-1 rather than cyclo-oxygenase-2 are associated with human gastrointestinal toxicity: A full in vitro analysis. *Proc. Natl. Acad. Sci. U.S.A.* 96 7563–7568. 10.1073/pnas.96.13.7563 10377455 PMC22126

[B45] Yoshida R. Ohkuri T. Jyotaki M. Yasuo T. Horio N. Yasumatsu K. (2010). Endocannabinoids selectively enhance sweet taste. *Proc. Natl. Acad. Sci. U.S.A.* 107 935–939. 10.1073/pnas.0912048107 20080779 PMC2818929

