# Supplementary material for: Cellular mechanisms of taste disturbance induced by the non-steroidal anti-inflammatory drug, diclofenac, in mice
Source: Front Cell Neurosci. 2023 Dec 18;17:1279059. doi: 10.3389/fncel.2023.1279059 (PMC10757961; doi:10.3389/fncel.2023.1279059)

Supplementary Figure 1.

Full length gel images of RT-PCR experiments for Figure 1.

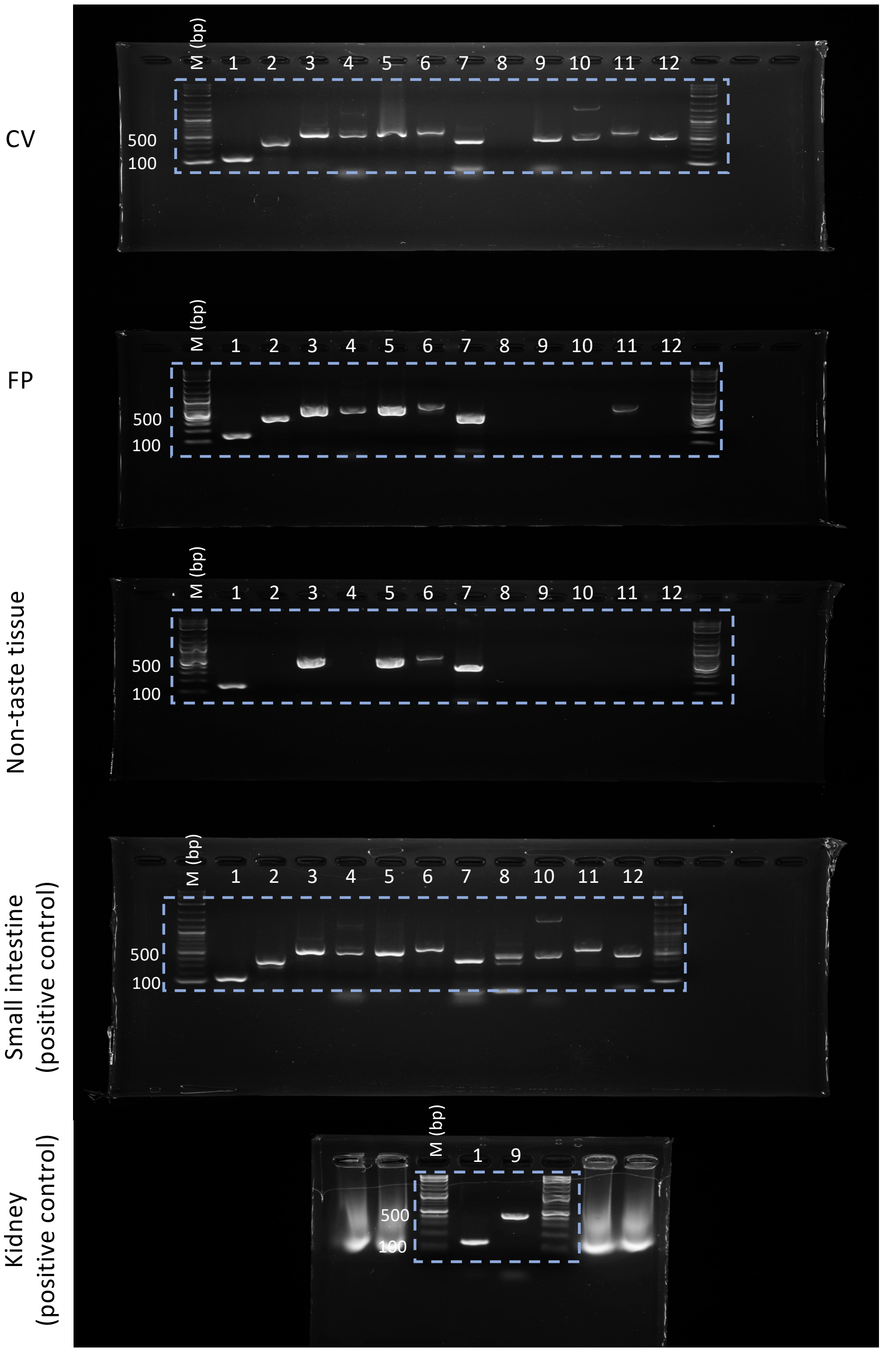

1	*Gapdh*
2	*Gustducin*
3	*Ptgs1(COX-1)*
4	*Ptgs2(COX-2)*
5	*mPges1*
6	*cPges*
7	*cPla2*
8	*sPla2*
9	*Pgds*
10	*Pgis*
11	*Prxl2b*
12	*Tbxas1*
	M(bp)

Reverse transcription-polymerase chain reaction was used to amplify the mRNAs for arachidonic acid pathway component molecules from the circumvallate papillae (CV), fungiform papillae (FP), tongue non-taste epithelial tissue, small intestine and lung (positive control for Arachidonic acid pathway components) of B6 mice. Areas enclosed by blue dot line were used in Figure 1. Arachidonic acid pathway components: prostaglandin-endoperoxide synthase 1/2(*Ptgs1/Ptgs2*), *), microsomal / cytosolic prostaglandin E synthase (mPges1/cPges)*, cytosolic/secretory phospholipase A2(*c/s Pla2*), prostaglandin D2 synthase (*Pgds*), prostaglandin I2 synthase (*Pgis*), peroxiredoxin like 2B (*Prxl2b*), thromboxane A synthase 1 (*Tbxas1*). *gustducin* (a taste cell marker), glyceraldehyde-3-phosphate dehydrogenase (*Gapdh*, housekeeping gene), M (bp) 100 bp marker ladder.

Supplementary Figure 2.

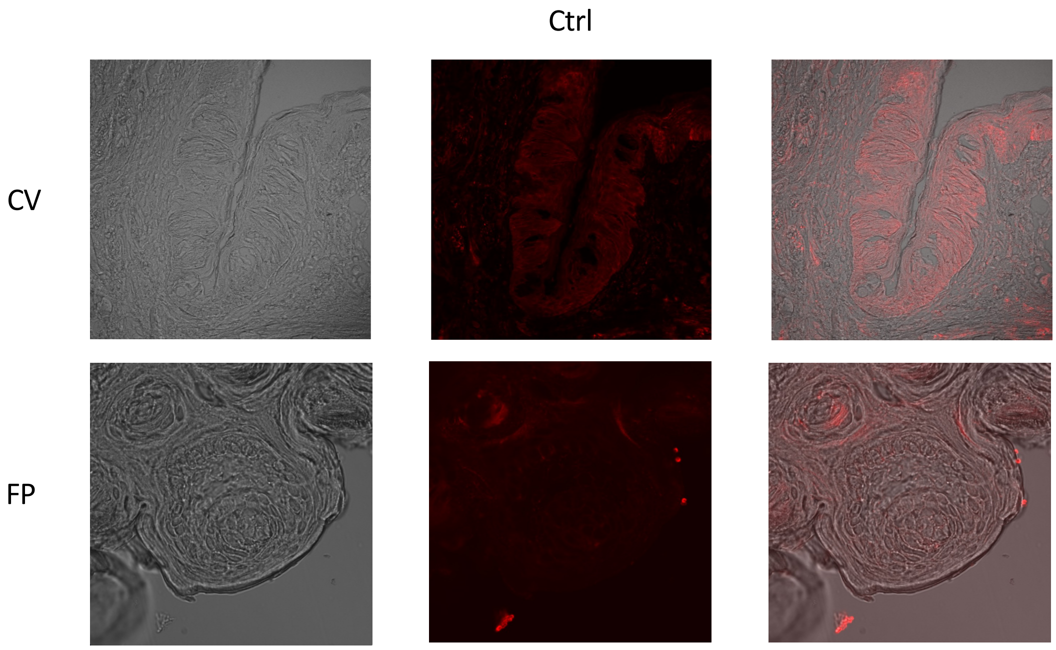

Images of immunohistochemical staining in the absence of primary antibodies for PTGS1 (COX-1) or cPGES in the circumvallate papillae (CV) and fungiform papillae (FP) of mice are shown as negative controls (red).

Supplementary Figure 3.

Comparison of sex differences in the number of positive cells per a taste bud in the immunohistochemical analyses (Figure 2,3). No significant differences in the number of positive cells were found between the sexes. (P > 0.05, Student's t-test).

		Taste papillae	Expression levels (male vs female)	Analysis	P value
COX1	Figure 2	CV	2.259 vs 2.279	Student's t-test	0.881
		FP	3.118 vs 3.300		0.439
cpges	Figure 3	CV	1.700 vs 1.680		0.8932
		FP	2.767 vs 2.704		0.810

Supplementary Figure 4.

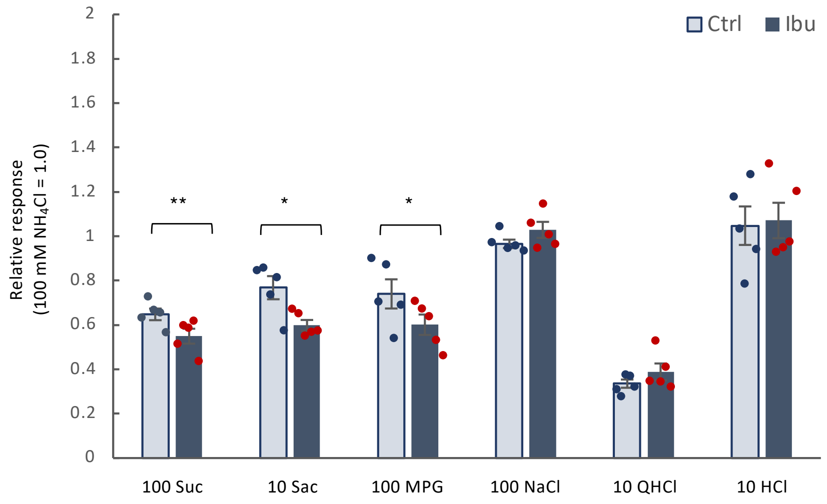

Figure	Analysis			P value
Supplementary Figure4.	paired t-test	Ctrl vs Ibu	100 mM Suc	< 0.01
			10 mM Sac	< 0.05
			100 mM MPG	< 0.05
			100 mM NaCl	0.258
			10 mM QHCl	0.348
			10 mM HCl	0.833

A single dose of ibuprofen Na (Ibu) affected chorda tympani (CT) nerve responses to sweet [sucrose (Suc) and saccharin (Sac)] and umami [monopotassium glutamate (MPG)]. Changes in CT nerve responses to 100 mM Suc, 10 mM Sac, 100 mM MPG, 100 mM NaCl, 10 mM QHCl and 10 mM HCl before (Ctrl) and 30-90 min after a single administration of ibuprofen Na (10-4 log M) were examined in mice (n = 5). The CT nerve responses to sweet (Suc and Sac) and umami (MPG) tastants were significantly reduced 30-90 min after ibuprofen Na administration, but not to NaCl, HCl and QHCl. The CT nerve responses are shown as mean ± SEM. *P < 0.05, **P < 0.01 (Ctrl vs. Ibuprofen Na; paired t-test).

Supplementary Figure 5.

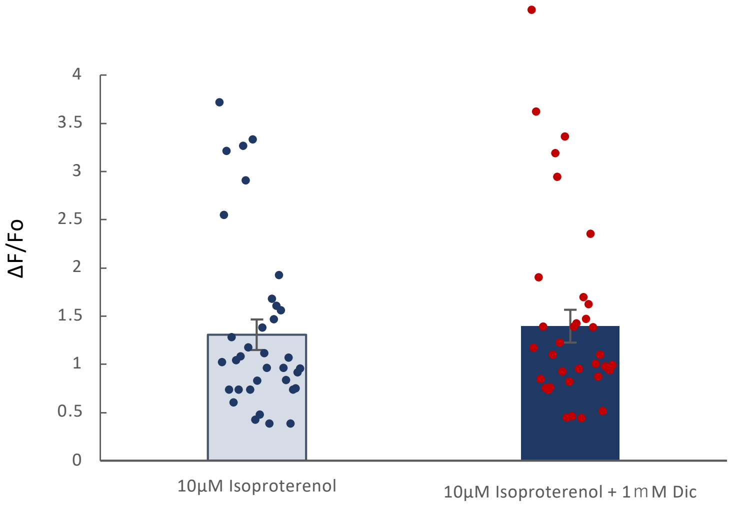

Figure	Analysis		P value
Supplementary Figure5.	paired t-test	10µM Isoproterenol vs 10µM Isoproterenol + 1mM Dic	0.1502

We tested whether diclofenac inhibits the activity of the endogenous beta-adrenergic receptor (belonging to the large family of G protein-coupled receptors, GPCRs) using Ca2+ imaging and a heterologous HEK293 cell expression system. We found that diclofenac did not inhibit the activation of the beta-adrenergic receptor by application of isoproterenol (10µM) in HEK293 cells ($P > 0.05$, paired t-test).

Supplementary Figure 6.
Relative change (Day1 = 1.0) in the body weight of the drug and control groups.

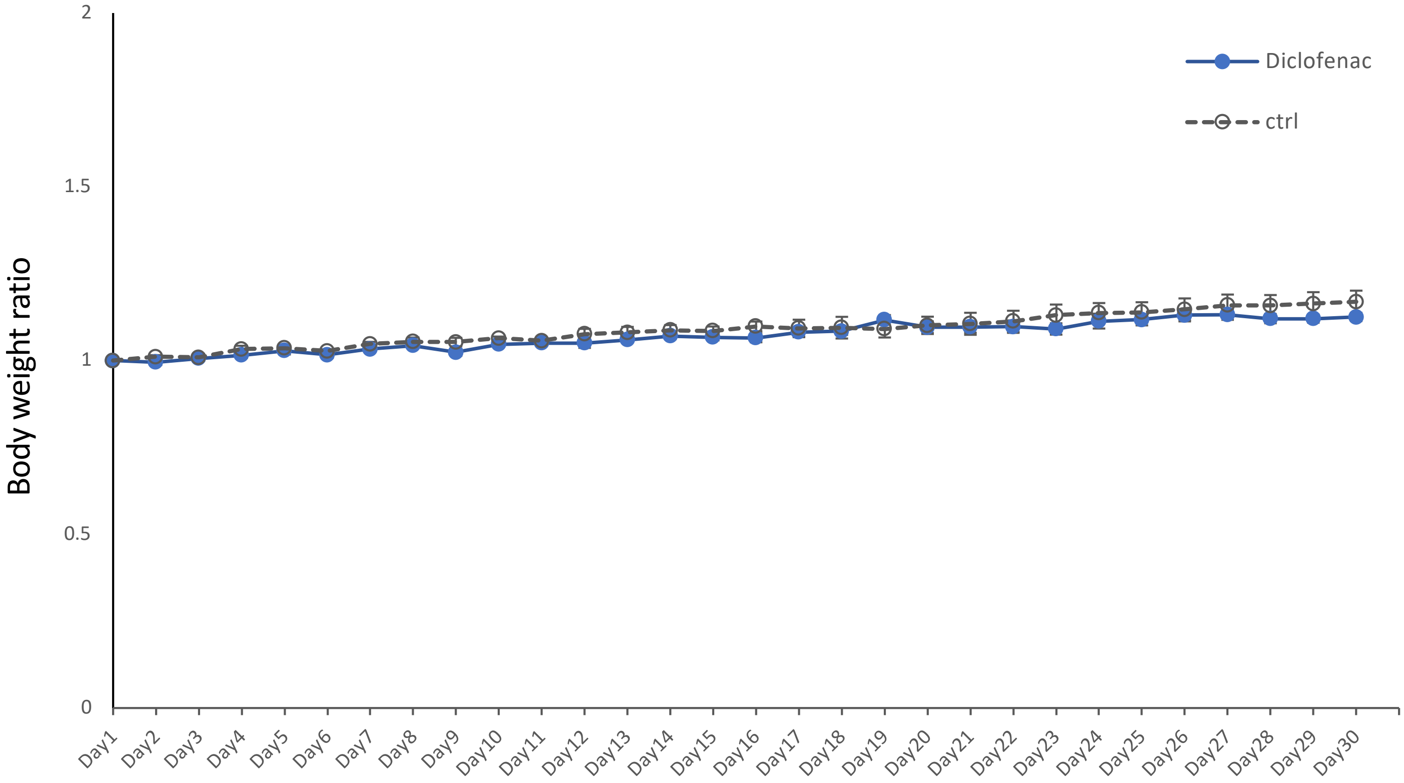

Supplement: Supplementary file 1 [file Data_Sheet_1.pdf]
