# Supplementary material for: Cellular mechanisms of taste disturbance induced by the non-steroidal anti-inflammatory drug, diclofenac, in mice
Source: Front Cell Neurosci. 2023 Dec 18;17:1279059. doi: 10.3389/fncel.2023.1279059 (PMC10757961; doi:10.3389/fncel.2023.1279059)
Supplement: Supplementary file 2 [file Data_Sheet_2.PDF]

<Ptgs1>

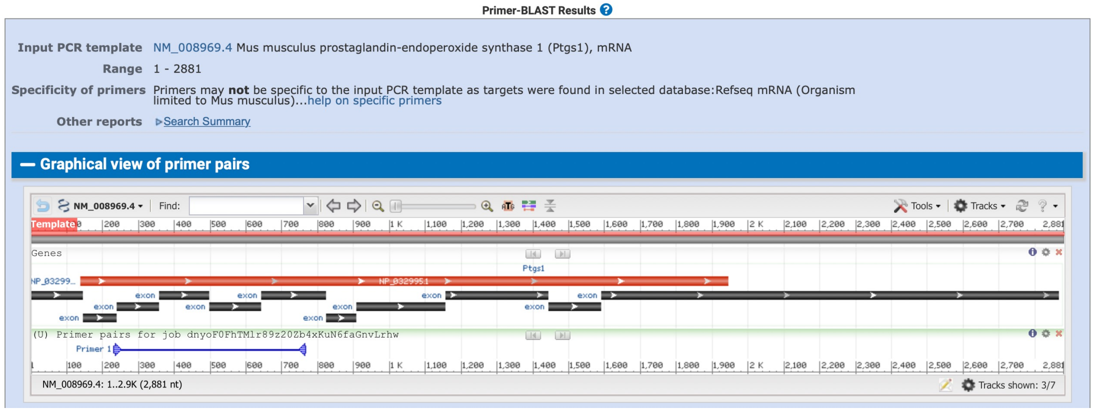

Mus musculus prostaglandin-endoperoxide synthase 1 (Ptgs1), mRNA

Sequence ID: NM_008969.4 **Length:** 2881 **Number of Matches:** 1

Range 1: 281 to 761 GenBank Graphics ▼ Next Match ▲ Previous Match

Score	Expect	Identities	Gaps	Strand
889 bits(481)	0.0	481/481(100%)	0/481(0%)	Plus/Plus

```
Query  1    CTGTGTCCGCTTTGGCCTCGACAACTACCAGTGTGATTGTACTCGCACGGGCTACTCAGG  60
            ||||||||||||||||||||||||||||||||||||||||||||||||||||||||||||
Sbjct  281  CTGTGTCCGCTTTGGCCTCGACAACTACCAGTGTGATTGTACTCGCACGGGCTACTCAGG  340

Query  61   CCCCAACTGTACCATCCCTGAGATCTGGACCTGGCTTCGGAATTCTCTGCGGCCCAGCCC  120
            ||||||||||||||||||||||||||||||||||||||||||||||||||||||||||||
Sbjct  341  CCCCAACTGTACCATCCCTGAGATCTGGACCTGGCTTCGGAATTCTCTGCGGCCCAGCCC  400

Query  121  CTCGTTCACCCATTTCCTGCTGACACATGGATACTGGCTCTGGGAATTTGTGAATGCCAC  180
            ||||||||||||||||||||||||||||||||||||||||||||||||||||||||||||
Sbjct  401  CTCGTTCACCCATTTCCTGCTGACACATGGATACTGGCTCTGGGAATTTGTGAATGCCAC  460

Query  181  CTTCATCCGAGAAGTACTCATGCGCCTGGTACTCACAGTGCGGTCCAACCTTATCCCCAG  240
            ||||||||||||||||||||||||||||||||||||||||||||||||||||||||||||
Sbjct  461  CTTCATCCGAGAAGTACTCATGCGCCTGGTACTCACAGTGCGGTCCAACCTTATCCCCAG  520

Query  241  CCCTCCGACCTACAACTCAGCGCATGACTACATCAGCTGGGAGTCCTTCTCCAATGTGAG  300
            ||||||||||||||||||||||||||||||||||||||||||||||||||||||||||||
Sbjct  521  CCCTCCGACCTACAACTCAGCGCATGACTACATCAGCTGGGAGTCCTTCTCCAATGTGAG  580

Query  301  CTACTATACTCGCATTCTGCCCTCTGTACCCAAAGACTGCCCCACACCCATGGGGACCAA  360
            ||||||||||||||||||||||||||||||||||||||||||||||||||||||||||||
Sbjct  581  CTACTATACTCGCATTCTGCCCTCTGTACCCAAAGACTGCCCCACACCCATGGGGACCAA  640

Query  361  AGGGAAGAAACAGTTACCAGATGTTCAGCTTCTGGCCCAACAGCTGCTGCTGAGAAGGGA  420
            ||||||||||||||||||||||||||||||||||||||||||||||||||||||||||||
Sbjct  641  AGGGAAGAAACAGTTACCAGATGTTCAGCTTCTGGCCCAACAGCTGCTGCTGAGAAGGGA  700

Query  421  GTTCATTCCTGCCCCCCAGGGCACCAACATCCTGTTTGCCTTCTTTGCACAACACTTCAC  480
            ||||||||||||||||||||||||||||||||||||||||||||||||||||||||||||
Sbjct  701  GTTCATTCCTGCCCCCCAGGGCACCAACATCCTGTTTGCCTTCTTTGCACAACACTTCAC  760

Query  481  C  481
            |
Sbjct  761  C  761
```

<Ptgs2>

Input PCR template NM_011198.5 Mus musculus prostaglandin-endoperoxide synthase 2 (Ptgs2), mRNA

Range 1 - 4384

Specificity of primers Primers may **not** be specific to the input PCR template as targets were found in selected database:Refseq mRNA (Organism limited to Mus musculus)...help on specific primers

Other reports ▷Search Summary

— Graphical view of primer pairs

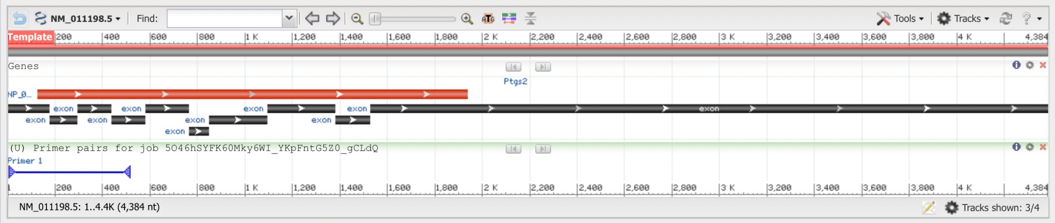

Mus musculus prostaglandin-endoperoxide synthase 2 (Ptgs2), mRNA

Sequence ID: NM_011198.5 Length: 4384 Number of Matches: 1

Range 1: 62 to 506 GenBank Graphics ▼ Next Match ▲ Previous Match

Score	Expect	Identities	Gaps	Strand
822 bits(445)	0.0	445/445(100%)	0/445(0%)	Plus/Plus

```
Query  1    TCAGCACTGCATCCTGCCAGCTCCACCGCCACCACTACTGCCACCTCCGCTGCCACCTCT  60
            ||||||||||||||||||||||||||||||||||||||||||||||||||||||||||||
Sbjct  62   TCAGCACTGCATCCTGCCAGCTCCACCGCCACCACTACTGCCACCTCCGCTGCCACCTCT  121

Query  61   GCGATGCTCTTCCGAGCTGTGCTGCTCTGCGCTGCCCTGGGGCTCAGCCAGGCAGCAAAT  120
            ||||||||||||||||||||||||||||||||||||||||||||||||||||||||||||
Sbjct  122  GCGATGCTCTTCCGAGCTGTGCTGCTCTGCGCTGCCCTGGGGCTCAGCCAGGCAGCAAAT  181

Query  121  CCTTGCTGTTCCAATCCATGTCAAAACCGTGGGGAATGTATGAGCACAGGATTTGACCAG  180
            ||||||||||||||||||||||||||||||||||||||||||||||||||||||||||||
Sbjct  182  CCTTGCTGTTCCAATCCATGTCAAAACCGTGGGGAATGTATGAGCACAGGATTTGACCAG  241

Query  181  TATAAGTGTGACTGTACCCGGACTGGATTCTATGGTGAAAACTGTACTACACCTGAATTT  240
            ||||||||||||||||||||||||||||||||||||||||||||||||||||||||||||
Sbjct  242  TATAAGTGTGACTGTACCCGGACTGGATTCTATGGTGAAAACTGTACTACACCTGAATTT  301

Query  241  CTGACAAGAATCAAATTACTGCTGAAGCCCACCCCAAACACAGTGCACTACATCCTGACC  300
            ||||||||||||||||||||||||||||||||||||||||||||||||||||||||||||
Sbjct  302  CTGACAAGAATCAAATTACTGCTGAAGCCCACCCCAAACACAGTGCACTACATCCTGACC  361

Query  301  CACTTCAAGGGAGTCTGGAACATTGTGAACAACATCCCCTTCCTGCGAAGTTTAATCATG  360
            ||||||||||||||||||||||||||||||||||||||||||||||||||||||||||||
Sbjct  362  CACTTCAAGGGAGTCTGGAACATTGTGAACAACATCCCCTTCCTGCGAAGTTTAATCATG  421

Query  361  AAATATGTGCTGACATCCAGATCATATTTGATTGACAGTCCACCTACTTACAATGTGCAC  420
            ||||||||||||||||||||||||||||||||||||||||||||||||||||||||||||
Sbjct  422  AAATATGTGCTGACATCCAGATCATATTTGATTGACAGTCCACCTACTTACAATGTGCAC  481

Query  421  TATGGTTACAAAAGCTGGGAAGCCT  445
            |||||||||||||||||||||||||
Sbjct  482  TATGGTTACAAAAGCTGGGAAGCCT  506
```

<mpges1>

Input PCR template NM_022415.3 Mus musculus prostaglandin E synthase (Ptges), mRNA

Range 1 - 3647

Specificity of primers Primers may **not** be specific to the input PCR template as targets were found in selected database:NCBI Transcript Reference Sequences (Organism limited to Mus musculus)...help on specific primers

Other reports ▷Search Summary

— Graphical view of primer pairs

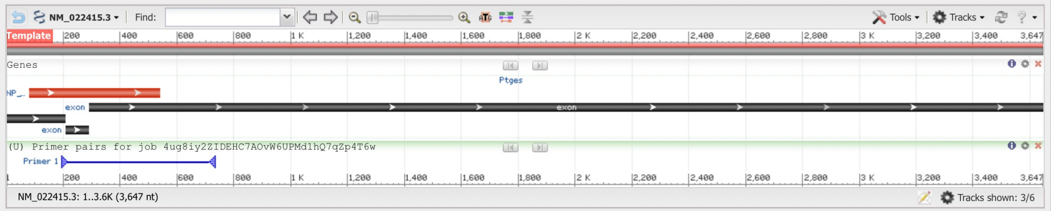

Mus musculus prostaglandin E synthase (Ptges), mRNA

Sequence ID: NM_022415.3 Length: 3647 Number of Matches: 1

Range 1: 242 to 718 GenBank Graphics ▼ Next Match ▲ Previous Match

Score	Expect	Identities	Gaps	Strand
881 bits(477)	0.0	477/477(100%)	0/477(0%)	Plus/Plus

```
Query  1    GGAGGCCTCCAGTATTACAGGAGTGACCCAGATGTGGAGCGCTGCCTCAGAGCCCACCGC  60
            ||||||||||||||||||||||||||||||||||||||||||||||||||||||||||||
Sbjct  242  GGAGGCCTCCAGTATTACAGGAGTGACCCAGATGTGGAGCGCTGCCTCAGAGCCCACCGC  301

Query  61   AACGACATGGAGACAATCTATCCTTTCCTCTTCCTCGGCTTCGTGTACTCATTCCTGGGA  120
            ||||||||||||||||||||||||||||||||||||||||||||||||||||||||||||
Sbjct  302  AACGACATGGAGACAATCTATCCTTTCCTCTTCCTCGGCTTCGTGTACTCATTCCTGGGA  361

Query  121  CCCAACCCTCTGATCGCCTGGATACATTTCCTCGTTGTCCTCACAGGCCGTGTGGTACAC  180
            ||||||||||||||||||||||||||||||||||||||||||||||||||||||||||||
Sbjct  362  CCCAACCCTCTGATCGCCTGGATACATTTCCTCGTTGTCCTCACAGGCCGTGTGGTACAC  421

Query  181  ACCGTGGCCTACCTGGGCAAGCTGAACCCACGCCTTCGCTCCGGGGCCTATGTCCTGGCC  240
            ||||||||||||||||||||||||||||||||||||||||||||||||||||||||||||
Sbjct  422  ACCGTGGCCTACCTGGGCAAGCTGAACCCACGCCTTCGCTCCGGGGCCTATGTCCTGGCC  481

Query  241  CAGTTCTCCTGTTTCTCCATGGCCCTGCAGATCCTCTGGGAGGTGGCCCACCATCTGTGA  300
            ||||||||||||||||||||||||||||||||||||||||||||||||||||||||||||
Sbjct  482  CAGTTCTCCTGTTTCTCCATGGCCCTGCAGATCCTCTGGGAGGTGGCCCACCATCTGTGA  541

Query  301  CCAGCAGCTGAAGCCTCCTCAGCTACCACAGTGGGGACAGAGCCATGAAGGCTGAACTTG  360
            ||||||||||||||||||||||||||||||||||||||||||||||||||||||||||||
Sbjct  542  CCAGCAGCTGAAGCCTCCTCAGCTACCACAGTGGGGACAGAGCCATGAAGGCTGAACTTG  601

Query  361  CGGACTTGGTGTCTCTTCTAGAATAGGGACGGGGTCTGGTCCTGGGGTCCTGGGTAACCT  420
            ||||||||||||||||||||||||||||||||||||||||||||||||||||||||||||
Sbjct  602  CGGACTTGGTGTCTCTTCTAGAATAGGGACGGGGTCTGGTCCTGGGGTCCTGGGTAACCT  661

Query  421  GGGCGAGTGGATCTCAGGGCCCAATGTGCACATGTGTGTTTCTTAGCCTTTTGGGAT  477
            |||||||||||||||||||||||||||||||||||||||||||||||||||||||||
Sbjct  662  GGGCGAGTGGATCTCAGGGCCCAATGTGCACATGTGTGTTTCTTAGCCTTTTGGGAT  718
```

<cpges>

Input PCR template NM_019766.4 Mus musculus prostaglandin E synthase 3 (Ptges3), mRNA
Range 1 - 1954
Specificity of primers Primer pairs are specific to input template as no other targets were found in selected database: NCBI Transcript Reference Sequences (Organism limited to Mus musculus)
Other reports ▷Search Summary

— Graphical view of primer pairs

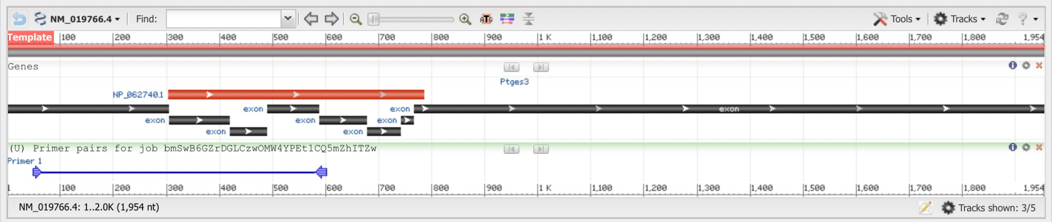

Mus musculus prostaglandin E synthase 3 (Ptges3), mRNA

Sequence ID: NM_019766.4 Length: 1954 Number of Matches: 1

Range 1: 98 to 190 GenBank Graphics ▼ Next Match ▲ Previous Match

Score	Expect	Identities	Gaps	Strand
167 bits(90)	2e-36	92/93(99%)	0/93(0%)	Plus/Plus

```
Query  1    TTCTCTCCCGGGTTGAGGGAGCCGCTCTCCGCGCGGTGCATTCTGGGCATCGAGGTCGAg  60
            ||||||||||||||||||||||||||||||||||||||||||||||||||||||| ||||
Sbjct  98   TTCTCTCCCGGGTTGAGGGAGCCGCTCTCCGCGCGGTGCATTCTGGGCATCGAGGCCGAG  157

Query  61   cccgccgccgccgtcgcctaagaggagcgagga  93
            |||||||||||||||||||||||||||||||||
Sbjct  158  CCCGCCGCCGCCGTCGCCTAAGAGGAGCGAGGA  190
```

<spla2>

Input PCR template NM_001082531.1 Mus musculus phospholipase A2, group IIA (platelets, synovial fluid) (Pla2g2a), transcript variant 1, coding, mRNA

Range 1 - 793

Specificity of primers Primers may **not** be specific to the input PCR template as targets were found in selected database:Refseq mRNA (Organism limited to Mus musculus)...help on specific primers

Other reports ▷Search Summary

— Graphical view of primer pairs

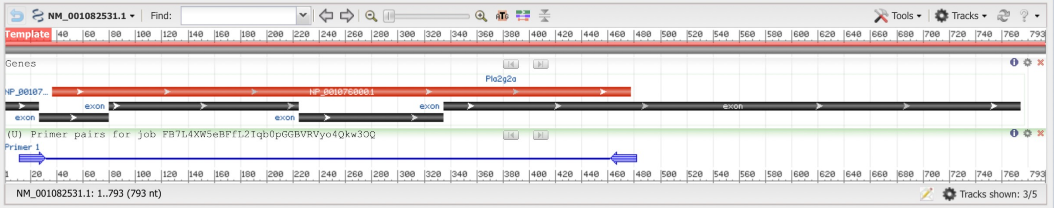

Mus musculus phospholipase A2, group IIA (platelets, synovial fluid) (Pla2g2a), transcript variant 1, coding, mRNA

Sequence ID: NM_001082531.1 **Length:** 793 **Number of Matches:** 1

See 1 more title(s) See all Identical Proteins(IPG)

Range 1: 62 to 441 GenBank Graphics ▼ Next Match ▲ Previous Match

Score	Expect	Identities	Gaps	Strand
686 bits(371)	0.0	378/381(99%)	1/381(0%)	Plus/Plus

```
Query  1    CCTCGATCATGGCCTTTGGCTCAATACAGGTCCAAGGGAACATTGCGCAGTTTGGGGAAA  60
            ||||||||||||||||||||||||||||||||||||||||||||||||||||||||||||
Sbjct  62   CCTCGATCATGGCCTTTGGCTCAATACAGGTCCAAGGGAACATTGCGCAGTTTGGGGAAA  121

Query  61   TGATTTGGCTTAAGACAGGAAAGAGAGCTGAGCTTAGCTATGCCTTCTATGGATGCCACT  120
            ||||| ||||||||||||||||||||||||||||||||||||||||||||||||||||||
Sbjct  122  TGATTCGGCTTAAGACAGGAAAGAGAGCTGAGCTTAGCTATGCCTTCTATGGATGCCACT  181

Query  121  GTGGCCTGGGTGGCAAAGGATTCCCCCAAGGATGCCACAGACCGGTGCTGTGTTACTCAT  180
            |||||||||||||||||||| |||||||||||||||||||||||||||||||||||||||
Sbjct  182  GTGGCCTGGGTGGCAAAGGA-TCCCCCAAGGATGCCACAGACCGGTGCTGTGTTACTCAT  240

Query  181  GACTGTTGCTACAAGCGCCTGGAGAAAAGTGGATGTGGTACTAAGTTACTGAAATACAAG  240
            ||||||||||||||| ||||||||||||||||||||||||||||||||||||||||||||
Sbjct  241  GACTGTTGCTACAAGAGCCTGGAGAAAAGTGGATGTGGTACTAAGTTACTGAAATACAAG  300

Query  241  TACTCCCACCAAGGGGGCCAAATCACCTGTTCTGCAAACCAGAACTCCTGTCAGAAACGG  300
            ||||||||||||||||||||||||||||||||||||||||||||||||||||||||||||
Sbjct  301  TACTCCCACCAAGGGGGCCAAATCACCTGTTCTGCAAACCAGAACTCCTGTCAGAAACGG  360

Query  301  CTGTGTCAGTGCGATAAAGCCGCCGCTGAATGTTTCGCCCGGAACAAGAAAACCTACAGT  360
            ||||||||||||||||||||||||||||||||||||||||||||||||||||||||||||
Sbjct  361  CTGTGTCAGTGCGATAAAGCCGCCGCTGAATGTTTCGCCCGGAACAAGAAAACCTACAGT  420

Query  361  TTAAAGTACCAGTTCTACCCC  381
            |||||||||||||||||||||
Sbjct  421  TTAAAGTACCAGTTCTACCCC  441
```

Related Information

Gene - associated gene details

GEO Profiles - microarray expression data

PubChem BioAssay - bioactivity screening

<cpla2>

Input PCR template NM_001305632.1 Mus musculus phospholipase A2, group IVA (cytosolic, calcium-dependent) (Pla2g4a), transcript variant 2, mRNA

Range 1 - 2866

Specificity of primers Primers may **not** be specific to the input PCR template as targets were found in selected database:Refseq mRNA (Organism limited to Mus musculus)...help on specific primers

Other reports ▷Search Summary

— Graphical view of primer pairs

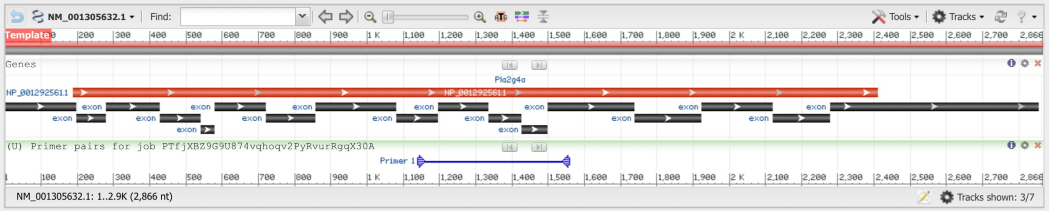

Mus musculus phospholipase A2, group IVA (cytosolic, calcium-dependent) (Pla2g4a), transcript variant 2, mRNA

Sequence ID: NM_001305632.1 **Length:** 2866 **Number of Matches:** 1

Range 1: 1190 to 1554 GenBank Graphics ▼ Next Match ▲ Previous Match

Score	Expect	Identities	Gaps	Strand
675 bits(365)	0.0	365/365(100%)	0/365(0%)	Plus/Plus

```
Query  1     TTTGCCGATTGGGTGGAATTTAGTCCATATGAGATTGGCATGGCAAAATATGGTACCTTT  60
             ||||||||||||||||||||||||||||||||||||||||||||||||||||||||||||
Sbjct  1190  TTTGCCGATTGGGTGGAATTTAGTCCATATGAGATTGGCATGGCAAAATATGGTACCTTT  1249

Query  61    ATGGCTCCTGACCTATTTGGAAGCAAGTTTTTTATGGGAACAGTTGTaaaaaaaTATGAA  120
             ||||||||||||||||||||||||||||||||||||||||||||||||||||||||||||
Sbjct  1250  ATGGCTCCTGACCTATTTGGAAGCAAGTTTTTTATGGGAACAGTTGTAAAAAAATATGAA  1309

Query  121   GAAAACCCCTTGCATTTCTTGATGGGTGTCTGGGGCAGTGCCTTTTCTATACTGTTCAAC  180
             ||||||||||||||||||||||||||||||||||||||||||||||||||||||||||||
Sbjct  1310  GAAAACCCCTTGCATTTCTTGATGGGTGTCTGGGGCAGTGCCTTTTCTATACTGTTCAAC  1369

Query  181   AGAGTTTTGGGAGTTTCTGGCTCACAGAATAAAGGCTCTACAATGGAAGAGGAATTAGAA  240
             ||||||||||||||||||||||||||||||||||||||||||||||||||||||||||||
Sbjct  1370  AGAGTTTTGGGAGTTTCTGGCTCACAGAATAAAGGCTCTACAATGGAAGAGGAATTAGAA  1429

Query  241   AATATTACAGCAAAGCACATCGTGAGTAATGACAGCTCCGACAGTGATGATGAGGCTCAA  300
             ||||||||||||||||||||||||||||||||||||||||||||||||||||||||||||
Sbjct  1430  AATATTACAGCAAAGCACATCGTGAGTAATGACAGCTCCGACAGTGATGATGAGGCTCAA  1489

Query  301   GGACCCAAAGGCACCGAGAATGAAGAAGCTGAAAAAGAGTACCAAAGCGACAACCAAGCA  360
             ||||||||||||||||||||||||||||||||||||||||||||||||||||||||||||
Sbjct  1490  GGACCCAAAGGCACCGAGAATGAAGAAGCTGAAAAAGAGTACCAAAGCGACAACCAAGCA  1549

Query  361   AGTTG  365
             |||||
Sbjct  1550  AGTTG  1554
```

Related Information

- Gene - associated gene details
- GEO Profiles - microarray expression data
- PubChem BioAssay - bioactivity screening
- Genome Data Viewer - aligned genomic context

<pgds>

Input PCR template NM_001420744.1 Mus musculus prostaglandin D2 synthase (brain) (Ptgds), transcript variant 2, mRNA

Range 1 - 850

Specificity of primers Primers may **not** be specific to the input PCR template as targets were found in selected database:Refseq mRNA (Organism limited to Mus musculus)...help on specific primers

Other reports ▹Search Summary

— Graphical view of primer pairs

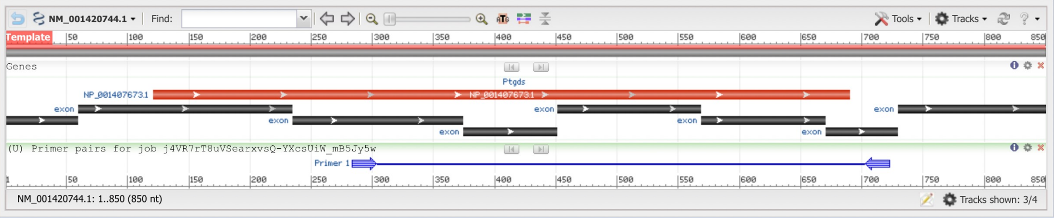

Mus musculus prostaglandin D2 synthase (brain) (Ptgds), transcript variant 2, mRNA

Sequence ID: NM_001420744.1 **Length:** 850 **Number of Matches:** 1

Range 1: 341 to 722 GenBank Graphics ▼ Next Match ▲ Previous Match

Score	Expect	Identities	Gaps	Strand
695 bits(376)	0.0	380/382(99%)	0/382(0%)	Plus/Plus

```
Query  1    AAGGCGGCCTCAATCTCACCTCTACCTTCCTCAGGAAAAACCAGTGTGAGACCAAGATCA  60
            ||||||||||||||||||||||||||||||||||||||||||||||||||||||||||||
Sbjct  341  AAGGCGGCCTCAATCTCACCTCTACCTTCCTCAGGAAAAACCAGTGTGAGACCAAGATCA  400

Query  61   TGGTACTGCAGCCTGCGGGGGCTCCTGGACACTACACCTACAGCAGCCCCCACTCGGGCA  120
            ||||||||||||||||||||||||||||||||||||||||||||||||||||||||||||
Sbjct  401  TGGTACTGCAGCCTGCGGGGGCTCCTGGACACTACACCTACAGCAGCCCCCACTCGGGCA  460

Query  121  GCATCCACTCCGTGTCAGTGGTGGAGGCCAACTATGACGAGTACGCTCTGCTATTCAGCA  180
            ||||||||||||||||||||||||||||||||||||||||||||||||||||||||||||
Sbjct  461  GCATCCACTCCGTGTCAGTGGTGGAGGCCAACTATGACGAGTACGCTCTGCTATTCAGCA  520

Query  181  GAGGCACCAAGGGCCCAGGCCAGGACTTCCGCATGGCCACCCTCTACAGCAGAACCCAGA  240
            ||||||||||||||||||||||||||||||||||||||||||||||||||||||||||||
Sbjct  521  GAGGCACCAAGGGCCCAGGCCAGGACTTCCGCATGGCCACCCTCTACAGCAGAACCCAGA  580

Query  241  CTCTGAAGGACGAGCTGAAGGAGAAATTCACCACCTTTAGCAAGGCCCAGGGCCTCACAG  300
            ||||||||||||||||||||||||||||||||||||||||||||||||||||||||||||
Sbjct  581  CTCTGAAGGACGAGCTGAAGGAGAAATTCACCACCTTTAGCAAGGCCCAGGGCCTCACAG  640

Query  301  AGGAGGACATTGTTTTCCTGCCCCAACCGGATAAGTGCATTCAAGAGTAAACGCAGGTGA  360
            ||||||||||||||||||||||||||||||||||||||||||||||||||||||||||||
Sbjct  641  AGGAGGACATTGTTTTCCTGCCCCAACCGGATAAGTGCATTCAAGAGTAAACGCAGGTGA  700

Query  361  GAGAAGTCAGTCGTAGGGCTGG  382
            ||||||||||||  ||||||||
Sbjct  701  GAGAAGTCAGTCAGAGGGCTGG  722
```

<pgis>

Input PCR template NM_001420752.1 Mus musculus prostaglandin I2 (prostacyclin) synthase (Ptgis), transcript variant 2, mRNA

Range 1 - 1772

Specificity of primers Primers may **not** be specific to the input PCR template as targets were found in selected database:Refseq mRNA (Organism limited to Mus musculus)...help on specific primers

Other reports ▷Search Summary

— Graphical view of primer pairs

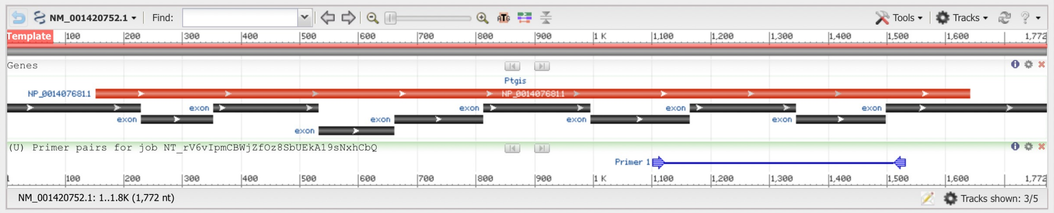

Mus musculus prostaglandin I2 (prostacyclin) synthase (Ptgis), transcript variant 2, mRNA

Sequence ID: NM_001420752.1 **Length:** 1772 **Number of Matches:** 1

Range 1: 1153 to 1530 GenBank Graphics ▼ Next Match ▲ Previous Match

Score	Expect	Identities	Gaps	Strand
699 bits(378)	0.0	378/378(100%)	0/378(0%)	Plus/Plus

```
Query  1     GCCTGTGCTAGACAGTGTGCTCAATGAGACACTCCGGCTCACGGCTGCCCCCTTCATCAC  60
             ||||||||||||||||||||||||||||||||||||||||||||||||||||||||||||
Sbjct  1153  GCCTGTGCTAGACAGTGTGCTCAATGAGACACTCCGGCTCACGGCTGCCCCCTTCATCAC  1212

Query  61    CCGTGAGGTCATGGCAGACCTGGCCTTGCCTATGGCAGACGGGAGGGAATTCTCTCTTCG  120
             ||||||||||||||||||||||||||||||||||||||||||||||||||||||||||||
Sbjct  1213  CCGTGAGGTCATGGCAGACCTGGCCTTGCCTATGGCAGACGGGAGGGAATTCTCTCTTCG  1272

Query  121   ACGTGGTGACCGCCTTCTCCTCTTTCCCTTCCTGAGTCCCCAGAAGGACCCAGAAATCTA  180
             ||||||||||||||||||||||||||||||||||||||||||||||||||||||||||||
Sbjct  1273  ACGTGGTGACCGCCTTCTCCTCTTTCCCTTCCTGAGTCCCCAGAAGGACCCAGAAATCTA  1332

Query  181   CACAGAGCCTGAGGTGTTTAAATACAACCGATTCTTGAACCCAGATGGATCTGAAAAGAA  240
             ||||||||||||||||||||||||||||||||||||||||||||||||||||||||||||
Sbjct  1333  CACAGAGCCTGAGGTGTTTAAATACAACCGATTCTTGAACCCAGATGGATCTGAAAAGAA  1392

Query  241   AGATTTTTACAAAGATGGGAAACGGCTGAAGAATTACAACATGCCATGGGGCGCAGGGCA  300
             ||||||||||||||||||||||||||||||||||||||||||||||||||||||||||||
Sbjct  1393  AGATTTTTACAAAGATGGGAAACGGCTGAAGAATTACAACATGCCATGGGGCGCAGGGCA  1452

Query  301   CAACCAGTGCCTGGGGAAGAGTTATGCCATCAACAGCATCAAACAATTTGTCGTCCTACT  360
             ||||||||||||||||||||||||||||||||||||||||||||||||||||||||||||
Sbjct  1453  CAACCAGTGCCTGGGGAAGAGTTATGCCATCAACAGCATCAAACAATTTGTCGTCCTACT  1512

Query  361   GCTGACTCATTTCGACCT  378
             ||||||||||||||||||
Sbjct  1513  GCTGACTCATTTCGACCT  1530
```

<prxl2b>

Input PCR template NM_025582.4 Mus musculus peroxiredoxin like 2B (Prxl2b), mRNA

Range 1 - 810

Specificity of primers Primers may **not** be specific to the input PCR template as targets were found in selected database:Refseq mRNA (Organism limited to Mus musculus)...help on specific primers

Other reports ▷Search Summary

— Graphical view of primer pairs

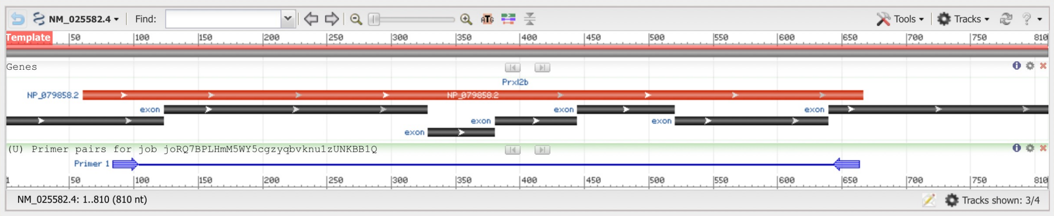

Mus musculus peroxiredoxin like 2B (Prxl2b), mRNA

Sequence ID: NM_025582.4 **Length:** 810 **Number of Matches:** 1

Range 1: 133 to 662 GenBank Graphics ▼ Next Match ▲ Previous Match

Score	Expect	Identities	Gaps	Strand
979 bits(530)	0.0	530/530(100%)	0/530(0%)	Plus/Plus

```
Query  1    CTGCGGAGCCTATGGCAGGAGAAAGCTTGTGTGGTGGCCGGTCTGCGACGCTTCGGCTGC  60
            ||||||||||||||||||||||||||||||||||||||||||||||||||||||||||||
Sbjct  133  CTGCGGAGCCTATGGCAGGAGAAAGCTTGTGTGGTGGCCGGTCTGCGACGCTTCGGCTGC  192

Query  61   ATGGTGTGCCGCTGGATCGCCCAGGACCTCAGCAACCTCCGGAGCATCCTGGACCAACAC  120
            ||||||||||||||||||||||||||||||||||||||||||||||||||||||||||||
Sbjct  193  ATGGTGTGCCGCTGGATCGCCCAGGACCTCAGCAACCTCCGGAGCATCCTGGACCAACAC  252

Query  121  GATGTGCGCCTGGTGGGCGTGGGGCCTGAGGCCCTGGGCCTGCAGGAGTTTCTGGATGGT  180
            ||||||||||||||||||||||||||||||||||||||||||||||||||||||||||||
Sbjct  253  GATGTGCGCCTGGTGGGCGTGGGGCCTGAGGCCCTGGGCCTGCAGGAGTTTCTGGATGGT  312

Query  181  GGTTACTTCTCAGGAGAACTCTACCTTGATGAGAGCAAGCAAATCTATAAGGAGCTGGGC  240
            ||||||||||||||||||||||||||||||||||||||||||||||||||||||||||||
Sbjct  313  GGTTACTTCTCAGGAGAACTCTACCTTGATGAGAGCAAGCAAATCTATAAGGAGCTGGGC  372

Query  241  TTCAAGCGGTACAACAGCTTAAGCATCCTACCAGCTGCCCTGGGAAAACCTGTTCGTGAT  300
            ||||||||||||||||||||||||||||||||||||||||||||||||||||||||||||
Sbjct  373  TTCAAGCGGTACAACAGCTTAAGCATCCTACCAGCTGCCCTGGGAAAACCTGTTCGTGAT  432

Query  301  GTAGCCTCCAAGGCTAAAGCTGTTGGTATCCAGGGGAACCTGTCTGGTGACCTGCTGCAA  360
            ||||||||||||||||||||||||||||||||||||||||||||||||||||||||||||
Sbjct  433  GTAGCCTCCAAGGCTAAAGCTGTTGGTATCCAGGGGAACCTGTCTGGTGACCTGCTGCAA  492

Query  361  AGTGGAGGGCTGCTGGTGGTCAGCAAGGGTGGCGACAAGGTACTGTTGCACTTCATCCAG  420
            ||||||||||||||||||||||||||||||||||||||||||||||||||||||||||||
Sbjct  493  AGTGGAGGGCTGCTGGTGGTCAGCAAGGGTGGCGACAAGGTACTGTTGCACTTCATCCAG  552

Query  421  AAGTCCCCGGGTGACTATGTTCCCCAGGAGAACATCCTGCAGGCCTTGGGTATCTCTGCA  480
            ||||||||||||||||||||||||||||||||||||||||||||||||||||||||||||
Sbjct  553  AAGTCCCCGGGTGACTATGTTCCCCAGGAGAACATCCTGCAGGCCTTGGGTATCTCTGCA  612

Query  481  GAGGTTTGCTCCAGCAAGCCACCCCAGTGTGATGAAGAGGTGTGTGGGAG  530
            ||||||||||||||||||||||||||||||||||||||||||||||||||
Sbjct  613  GAGGTTTGCTCCAGCAAGCCACCCCAGTGTGATGAAGAGGTGTGTGGGAG  662
```

<Tbxas1>

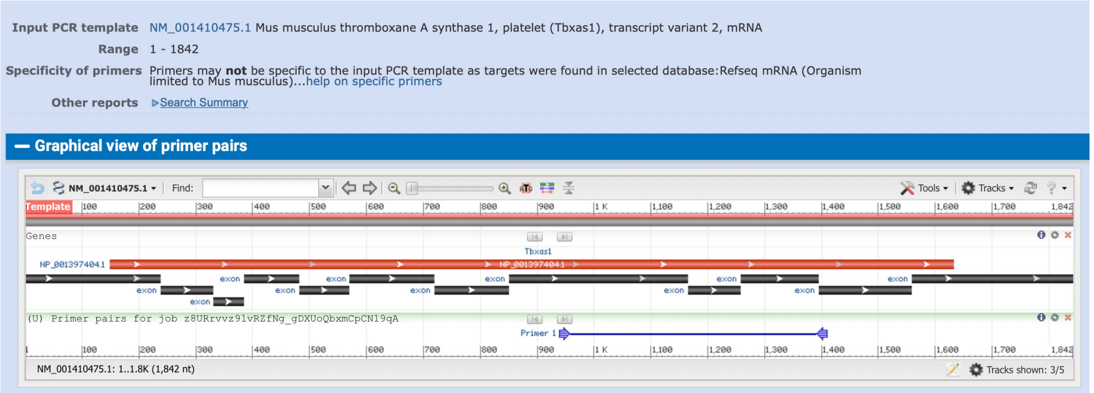

Mus musculus thromboxane A synthase 1, platelet (Tbxas1), transcript variant 2, mRNA

Sequence ID: NM_001410475.1 **Length:** 1842 **Number of Matches:** 1

Range 1: 987 to 1407 GenBank Graphics Next Match Previous Match

Score	Expect	Identities	Gaps	Strand
765 bits(414)	0.0	419/421(99%)	1/421(0%)	Plus/Plus

```
Query  1     CTACCT-CACATCTAAGCCTTTCACTGTGGATGAAATTGTGGGCCAGGCCTTCCTCTTCC  59
             |||||| ||||||||||||||||||||||||||||||||||||||||||||||||||||
Sbjct  987   CTACCTCCACATCTAAGCCTTTCACTGTGGATGAAATTGTGGGCCAGGCCTTCCTCTTCC  1046

Query  60    TCATTGCGGGCCATGAGGTCATCACAAACACGCTGTCCTTCATCACATACCTGCTGGCCA  119
             ||||||||||||||||||||||||||||||||||||||||||||||||||||||||||||
Sbjct  1047  TCATTGCGGGCCATGAGGTCATCACAAACACGCTGTCCTTCATCACATACCTGCTGGCCA  1106

Query  120   CCCACCCTGACTGCCAGGAGAGGCTTCTGAAAGAGGTGGACCTCTTCATGGGGAAGCACC  179
             ||||||||||||||||||||||||||||||||||||||||||||||||||||||||||||
Sbjct  1107  CCCACCCTGACTGCCAGGAGAGGCTTCTGAAAGAGGTGGACCTCTTCATGGGGAAGCACC  1166

Query  180   CAGCCCCTGAGTACCACAGCCTGCAGGAAGGTCTGCCGTATCTGGACATGGTGATTTCAG  239
             ||||||||||||||||||||||||||||||||||||||||||||||||||||||||||||
Sbjct  1167  CAGCCCCTGAGTACCACAGCCTGCAGGAAGGTCTGCCGTATCTGGACATGGTGATTTCAG  1226

Query  240   AGACCCTGAGGATGTACCCACCAGCTTTCAGGTTCACACGGGAGGCAGCACAGGACTGTG  299
             ||||||||||||||||||||||||||||||||||||||||||||||||||||||||||||
Sbjct  1227  AGACCCTGAGGATGTACCCACCAGCTTTCAGGTTCACACGGGAGGCAGCACAGGACTGTG  1286

Query  300   AGGTGCTGGGACAACGTATCCCTGCAGGTACAGTGCTGGAGATAGCTGTGGGTGCCCTAC  359
             ||||||||||||||||||||||||||||||||||||||||||||||||||||||||||||
Sbjct  1287  AGGTGCTGGGACAACGTATCCCTGCAGGTACAGTGCTGGAGATAGCTGTGGGTGCCCTAC  1346

Query  360   ACCATGACCCGGAGCACTGGCCGAATCCTGAGACCTTTGACCCTGAAAGGTTCACAGCAG  419
             |||||||||| |||||||||||||||||||||||||||||||||||||||||||||||||
Sbjct  1347  ACCATGACCCAGAGCACTGGCCGAATCCTGAGACCTTTGACCCTGAAAGGTTCACAGCAG  1406

Query  420   A  420
             |
Sbjct  1407  A  1407
```
